# Gut microbiota response to in vitro transit time variation is mediated by microbial growth rates, nutrient use efficiency and adaptation to in vivo transit time

**DOI:** 10.1186/s40168-023-01691-y

**Published:** 2023-11-06

**Authors:** Yorick Minnebo, Karen Delbaere, Valerie Goethals, Jeroen Raes, Tom Van de Wiele, Kim De Paepe

**Affiliations:** 1 Center for Microbial Ecology and Technology, Department of Biotechnology, Ghent University, Coupure Links 653, 9000 Ghent, Belgium; 2 Laboratory of Molecular Bacteriology, Department of Microbiology and Immunology, KU Leuven, Herestraat 49, 3000 Leuven, Belgium; 3 Center for Microbiology, VIB, Herestraat 49, 3000 Leuven, Belgium

**Keywords:** Personalised gut microbiome research, Gut microbial ecology, SHIME in vitro gut simulator, Gut retention time, Gut residence time, Gastrointestinal transit, Quantitative microbiome profiling

## Abstract

**Background:**

Transit time is an important modulator of the human gut microbiome. The inability to modify transit time as the sole variable hampers mechanistic in vivo microbiome research. We singled out gut transit time in an unprecedented in vitro approach by subjecting faecal microbial communities from six individuals with either short, medium or long in vivo transit times, to three different colonic transit times of 21, 32 and 63 h in the validated human gut in vitro model, SHIME.

**Results:**

Transit time was identified as the single most important driver of microbial cell concentrations (52%), metabolic activity (45%) and quantitative (24%) and proportional (22%) community composition. Deceleration of transit was characterised by a significant decrease of specific *Bifidobacterium* and *Veillonella* spp. and increase of specific fibre degrading bacteria and nutrient specialists, such as *Bacteroides*, *Prevotella*, *Ruminococcus*, *Bilophila* and *Akkermansia* spp. These microbial communities reached a higher population density and net carbohydrate fermentation, leading to an increased SCFA production at longer transit times. In contrast, the carbohydrate-to-biomass production efficiency was increased at shorter transits, particularly in well-adapted faecal microbiomes from donors with short in vivo transit. Said adaptation was also reflected in the carbohydrate-to-SCFA conversion efficiency which varied with donor, but also colon region and SCFA chain length. A long transit time promoted propionate production, whereas butyrate production and butyrate producers were selectively enriched in the proximal colon at medium transit time.

**Conclusion:**

Microbial growth rates and nutrient utilisation efficiency mediate the species-specific gut microbiota response to in vitro transit time variation, which is the main driver of in vitro microbial load, metabolism and community composition. Given the in vivo transit time variation within and between individuals, the personalisation of in vitro transit time based on in vivo data is required to accurately study intra- and inter-individual differences in gut microbiome structure, functionality and interactions with host and environmental modulators.

Video Abstract

**Supplementary Information:**

The online version contains supplementary material available at 10.1186/s40168-023-01691-y.

## Background

The human gut microbiome, a diverse microbial ecosystem comprising approximately 10^13^ microbial cells, is widely studied with a focus on the interplay with diet and health [1]. Besides diet, gut transit time—the time it takes for food to pass through the human gut—has recently been identified as a modulator of the gut microbiome [2–4]. Stool consistency is often used as a proxy for transit time, with increasing stool firmness corresponding to slower transits as a consequence of a prolonged water absorption in the colon [5]. The correlation between transit time and stool consistency was confirmed in in vivo population studies, pinpointing both transit time and stool consistency as the predominant factors modulating microbial load, metabolism, richness, diversity and community composition [2, 3, 6, 7]. More specifically, stool consistency was identified as the single most important non-redundant covariate, explaining more than 4% of the quantitative and 7% of the relative faecal microbiome composition [2, 8].

Transit time varies within and between individuals, thus contributing to intra- and inter-individual differences in the human gut microbiome [6]. Changes in intestinal transit time have been linked to microbiome-related diseases, such as small intestinal bacterial overgrowth, irritable bowel syndrome, obesity, diabetes, Parkinson’s and Alzheimer’s disease, inflammatory bowel disease, ulcerative colitis and colon cancer [9]. This highlights the significance of studying transit time [10, 11]. Yet, mechanistic studies in a quantitative setting are lacking.

In vivo mechanistic intervention studies are particularly challenging given the fact that transit time is intrinsic to an individual’s gut physiology and cannot be regulated in vivo without introducing confounding effects. Attempts to modify transit time with polyethylene glycol in animal studies proved very invasive and bulking agents (e.g. wheat bran) that accelerate gastrointestinal transit have a direct impact on the microbiome [12, 13]. Besides, transit time is influenced by a number of host and environmental factors which may also directly affect the gut microbiome. Observational in vivo research investigating the gut microbiome in relation to transit time is, therefore, confounded by a large number of hard-to-control variables such as diet, lifestyle, sex, age, body mass index, health, colonic anatomy, pH, gut hormones, bile acid metabolism, host physical activity and genetics [4, 9, 14–19]. Sampling is moreover constrained by practical challenges and ethical considerations, restricting the temporal and spatial resolution, thereby precluding the study of microbiome dynamics, niche diversification and spatial organisation [6, 7, 20–23]. In vitro models overcome these limitations and the reduced complexity permits the study of transit time at a high level of control, abstracting possible confounding effects.

We used the validated in vitro Simulator of the Human Intestinal Microbial Ecosystem (SHIME) to single out transit time as a driver of the quantitative and proportional shifts in the simulated gut microbiota derived from six human donors. Donors were selected based on a pre-screening of in vivo transit time. Faecal microbiomes from long, medium and short transit time donors were subsequently subjected to transit time variation in the SHIME.

## Methods

### Experimental set-up

The Simulator of the Human Intestinal Microbial Ecosystem (SHIME®, Prodigest, Zwijnaarde, Belgium), a dynamic in vitro gut model, was used to assess the effects of transit time on the (quantitative) microbial community composition and metabolism. The SHIME consisted of a simulated stomach and small intestine, a proximal and a distal colon compartment with a controlled temperature (37°C), mixing (200rpm) and diet (Figure S1A) [24, 25]. The proximal and distal colon pH of 5.6–5.9, respectively, 6.6–6.9, were maintained with built-in pH controllers and pumps regulating the dosage of 0.5M NaOH and HCl (Chem Lab, Zedelgem, Belgium). Residence or transit time was studied as a variable. Three different total colonic transit times categorised as short (ST, 21h), medium (MT, 32h) and long (LT, 63h) were evaluated. Transit time variation was implemented by adjusting the volumes of the colon compartments to the influent dosage rate (200mL 8h^−1^) multiplied with the corresponding transit time (Figure S1B).

### SHIME inoculation and operation

Six parallel SHIME systems were inoculated with six non-pooled faecal inocula, derived from six individuals without diagnosed diseases and without a history of antibiotic intake within 6 months prior to their donation (Figure S1C). Donors were selected based on their in vivo transit time which was estimated by consuming cooked corn (250g) and measuring the time between consumption and defaecation [17, 26]. Donor stratification into a short (20 ± 1h), medium (38.5 ± 4.5h) or long (68 ± 7h) in vivo transit time category was confirmed by the self-assessed stool frequency and assessment of the Bristol Stool Scale (BSS) of the fresh faecal samples by the authors (Table S1, Note S1).

Faeces were deposited in airtight containers, comprising an AnaeroGen™ sachet (Oxoid, Hampshire, UK) to generate an anaerobic environment. A faecal slurry was prepared by homogenising and diluting 20g of fresh human faeces in 100mL 0.1M phosphate buffer pH 6.8 (8.8g L^−1^ K_2_HPO_4_, 6.8g L^−1^ KH_2_PO_4_), containing 1g L^−1^ sodium thioglycolate as a reducing agent. To remove particulate matter, the homogenate was centrifuged (3min, 500*g*), and the supernatant was used as inoculum [27]. All colon vessels were immediately flushed with N_2_ for 10min to obtain anaerobic conditions (Air Liquide, Paris, France). The colon vessels were flushed only once at the start of the experiment and remained unopened in an airtight micro-anaerobic state for the remainder of the experiment [28]. The anaerobic state was verified with a Compact GC (Global Analyser Solutions, Breda, The Netherlands), equipped with a Molsieve 5A pre-column and Porabond column (CH_4_, O_2_, H_2_ and N_2_) and a thermal conductivity detector [28]. After inoculation and flushing, the system was left stagnant overnight (without flow-through).

The following morning, semi-continuous feeding was initiated and repeated in 8-h cycles (Figure S1A). Concentrated (1.5 ×) standardised nutritional SHIME medium (17.4g L^−1^ adult L-SHIME growth medium and 6g L^−1^ corn starch, ProDigest, Zwijnaarde, Belgium, acidified to pH 2 with 37% HCl) and pancreatic juice (Table S2) were pumped from a fridge (4°C) to the combined stomach- and small intestine vessel, which was flushed at the start and end of every feeding cycle (Figure S1A). To eliminate confounding effects originating from differences in nutrient concentrations, the proximal colon influent composition was modified in order to provide an equal volumetric nutrient loading rate for every transit time configuration (Table S3, 16.38g L_reactor_^−1^ day^−1^). Next, the stomach and small intestine content was transferred to the proximal colon, and subsequently distal colon. The SHIME microbial community stabilised after minimally 9 distal colon and 6 total colonic transits, which were completed after 6, 11 and 16 days for the short, medium and long transit configurations, respectively (Figure S1C). At the end of the stabilisation phase, no major short-chain fatty acid (SCFA) fluctuations were observed (Figure S2). Once a stable community was reached, the set-up ran for an additional five-day experimental phase, during which both the proximal and distal colon compartment were daily sampled anaerobically to follow up microbial cell, metabolite and carbohydrate concentrations. Samples were aliquoted to perform chemical and molecular analyses. Replicate measurements were performed on separate aliquots.

### Chemical and molecular analyses

#### Flow cytometry

Total microbial cell concentrations were analysed with flow cytometry according to Van Nevel et al. [29]. The samples were diluted 10^4^ times with 0.22μm filtered sterile anaerobic PBS (Table S4), and incubated with a viability staining mix (1% v/v) for 20min at 37℃. The used staining mix consisted of 10μL 10,000 × SYBR® Green I nucleic acid stain (Fisher Scientific, Merelbeke, Belgium) combined with propidium iodide (20μL 20mM propidium iodide, Fisher Scientific, Merelbeke, Belgium) in 0.22μm-filtered dimethyl sulfoxide (Sigma Aldrich, St. Louis, MO, USA). The incubated samples were immediately measured, in duplicate with a CS&T calibrated FACSVerse™ volumetric flow cytometer (BD Biosciences, Erembodegem, Belgium), equipped with a blue (488nm) laser and green (530/30nm) and red (> 670nm) emission detector compatible with the applied viability staining procedure. Microbial cell counts were determined through gating of the green and red fluorescence emission channels (Figure S3) and divided by the acquisition volume. Heat-killed samples (90℃ for 15min) were included to differentiate between intact and damaged microbial cell populations. Additionally, 0.22μm-filtered samples were included as negative controls to identify noise.

#### SCFA analysis

SCFA concentrations were determined after diethyl ether extraction followed by capillary gas chromatography (GC-2014, Shimadzu®, The Netherlands), using a DB-FFAP 123–3232 column (30m × 0.32mm × 0.25μm; Agilent, Belgium) and a flame ionisation detector as described by De Paepe et al. [30]. In short, 2mL of the sample was conditioned with 0.5mL sulphuric acid, 0.4g sodium chloride and 0.4mL internal standard (2-methyl hexanoic acid), to which 2mL diethyl ether was added. After centrifuging the mixture for 3min at 3000rpm, the top layer (ether) was transferred into a GC vial. Next, 1μL of sample was injected in the chromatograph.

#### Ammonium and lactate analysis

The colon compartments were additionally sampled the first day after inoculation (before initiating semi-continuous flow-through), after 4.5 transits (halfway stabilisation phase) and after 9 distal colon transits (stabilised community) to analyse ammonium and lactate concentrations.

Samples for ammonium and lactate analyses were filtered through a 0.22μm filter and diluted with Milli-Q water (Merck Millipore, Overijse, Belgium). Ammonium samples were diluted 20 times and analysed with a 761 Compact Ion Chromatograph (Metrohm, Switzerland) equipped with a conductivity detector. Lactate samples were diluted 5 times and measured with a 930 Compact IC Flex (Metrohm, Switzerland) with inline bicarbonate removal and a conductivity detector [28].

#### Colorimetric quantification of carbohydrates

Carbohydrate concentrations were determined colorimetrically in triplicate according to Josefsson (1976) with D-glucose (≥ 99.5%, Carl Roth, Karlsruhe, Germany) as a standard [31].

#### DNA-extraction and 16S rRNA gene amplicon sequencing

Aliquots of the faecal inoculum and stabilised samples were centrifuged (10min at 5000*g)* and stored at − 20℃ for DNA-extraction followed by next-generation 16S rRNA gene amplicon sequencing of the V4 region [8]. Sequencing of the SHIME and control samples (i.e. blanks, negative controls, positive controls with a known composition and a pure *Runella slithyformis* culture) was performed on an Illumina MiSeq platform (Illumina, Hayward, CA, USA) using Illumina MiSeq v2 chemistry at the VIB Nucleomics core (VIB, Gasthuisberg Campus, Leuven, Belgium). The V4 region of the 16S rRNA gene was amplified by PCR using primers (515F GTGYCAGCMGCCGCGGTAA and 806R GGACTACNVGGGTWTCTAAT) according to Vandeputte et al. [8].

#### Normalising metabolite and biomass production and carbohydrate utilisation

The net daily metabolite production rate (mmol day^−1^) was calculated by multiplying the differences between incoming and measured metabolite concentrations (mM) with the daily flow rate of 600 mL day^−1^, as described by De Paepe et al. [13]. In the distal colon, the metabolite concentrations from the proximal colon were subtracted [13]. The net biomass production rate (cells day^−1^) and net carbohydrate utilisation rate (g day^−1^) were calculated in a similar manner.

The net carbohydrate-to-metabolite production efficiency, i.e. net daily metabolite production relative to the net carbohydrate utilisation rate (mmol g^−1^), was calculated by dividing the net daily metabolite production rate (mmol day^−1^) by the net carbohydrate utilisation rate (g day^−1^). The nutrient-to-biomass conversion efficiency (cells g^−1^), i.e. the net biomass production rate relative to the net carbohydrate utilisation rate, was calculated analogously. In both proximal and distal colon, the metabolite production was also normalised to the biomass amount (mmol cells^−1^) by dividing the net daily metabolite production rate (mmol day^−1^) by the daily net biomass production rate (cells day^−1^).

### Bioinformatics and statistics

All further data processing, visualisations and statistical analyses were performed in R version 4.4.1 [32]. All data was visualised with ggplot2_3.3.5 and ggpubr_0.4.0 unless mentioned otherwise [33, 34].

The acquired flow cytometry fcs files were processed with the Phenoflow package to determine the total, intact and propidium iodide stained damaged cell counts and concentrations (Phenoflow_1.1.2) [35].

The amplicon data was processed with the mothur software package (v.1.42.3) as extensively discussed by De Paepe et al. and classified with the RDP 16S rRNA training set 16 [30, 36]. The top 25 most abundant operational taxonomic units (OTUs) and OTUs with a proportional presence of more than 5% within genera that responded significantly to transit time (Table S5) were classified at species level using both the RDP SeqMatch tool (type strain, near-full-length and good quality sequences) with nomenclatural taxonomy and NCBI BLAST (highly similar sequences) (accession: December 2021). In the event of inconsistencies in the results of the RDP SeqMatch tool and NCBI BLAST, no species level classification is provided (Table S6).

The mothur processed amplicon sequencing data, consisting of a read count table (containing the number of reads observed for each OTU in each sample) and the taxonomic annotation, were subjected to quality control and further processed in R version 4.4.1 (2021–08-10). Singletons and OTUs present in less than 5% of the samples or with read counts not exceeding 0.5 times the number of samples were removed [37]. The read counts, proportional microbial community composition and reproducibility of the control samples were satisfactory (Figure S4). Rarefaction curves of all samples were additionally constructed to ensure sufficient sequencing depths (Figure S5) (vegan_2.5–7) [38]. Sample 121 (donor 2, distal colon, short SHIME transit, day 7) was removed from the dataset, as it had a read count (83) in the range of the blanks and negative controls (max 748 reads). The proportional composition of the other samples was inspected with phyloseq (v 1.36.0) at genus and OTU level [37].

Quantitative microbial profiles (QMP) were generated by combining flow cytometry cell concentrations and proportional microbial profiles (PMP) determined through 16S rRNA gene amplicon sequencing. The quantitative microbial community composition was calculated by multiplying flow cytometry total microbial cell concentrations (Phenoflow_1.1.2) with proportional, copy-number-corrected (using the RDP classifier tool, RDP 16S rRNA training set 16) rarefied mothur-processed 16S rRNA gene read counts [8, 35]. Principal coordinates analyses (PCoA) based on the Bray–Curtis dissimilarity measure were visualised on genus and OTU-level to explore both the quantitative and proportional microbial community variation relating to different SHIME transit times (phyloseq_1.36.0) [33, 37].

Kruskal–Wallis tests were performed to determine statistically significant differences between SCFA concentrations, total and intact microbial cell concentrations and quantitative and proportional taxon abundances between SHIME transit times and colon compartments (stats_3.6.3) [32]. Subsequently, post hoc tests with Holm correction were used to compare SHIME transit times (unpaired two-sided Wilcoxon signed rank test) and proximal and distal colon compartment for each SHIME transit time (paired two-sided Wilcoxon signed rank tests) (stats_3.6.3), (ggpubr_0.4.0) [32, 34]. Donors without genus or OTU specific quantitative or proportional taxon abundances in at least one SHIME transit were excluded for the respective genus or OTU comparison. Benjamini–Hochberg multiple testing corrections were performed and the resulting P_FDR_ values were displayed where applicable (stats_4.2.1).

Distance-based redundancy analyses (db-RDA) were performed based on the Bray–Curtis dissimilarity and visualised in a type 2 scaling correlation triplot to assess the significance of the SHIME transit time, donor, in vivo transit time and colon region constraints on the absolute and proportional net metabolite concentrations, total microbial cell concentrations, percentage intact microbial cells, and quantitative and proportional microbial community compositions (vegan_2.5–7) [38]. A Holm correction was applied for multiple testing (stats_3.6.2). Due to multicollinearity between donor and in vivo transit time, two separate models were built with either the factor donor or the factor in vivo transit time as independent variable, next to SHIME transit time and colon region (vegan_2.5–7) [38]. Similar results were obtained for the two different models using either donor or in vivo transit time as covariate and SHIME transit time or colon region as explanatory variable. The constrained fractions of the variance were adjusted by applying a subtractive procedure (*R*^2^_adjusted_) and depicted in the top right corners of each db-RDA plot [39]. The two first canonical axes were annotated with the proportional constrained eigenvalues. Site scores were displayed as weighed sums of species scores and the factor levels of explanatory variables were represented as centroids.

Integrative analysis of the quantitative genus level community composition and net daily metabolite production rate was performed with sparse partial least squares (sPLS) analysis from the mixOmics (6.18.1) package [40]. In short, a basic sPLS model was created in regression mode with the absolute genus level abundance data explaining the net SCFA production data. This model was then tuned through the extraction of the optimal number of variables (comp1 = 15, comp2 and 3 = 30 for the absolute genus level abundance data and comp1, 2 and 3 = 3 for the net SCFA production) and components (*n* = 3) in regression mode with correlation evaluation. These parameters were implemented for the construction of the final sPLS model [40]. Relevance network graphs, to assess structure associations between variables, were visualised with igraph_1.2.11 and mixOmics_6.18.1 [40, 41].

## Results

### Decelerating SHIME transit significantly increased microbial cell concentrations and net carbohydrate utilisation but significantly decreased biomass production efficiency

**Fig. 1:**
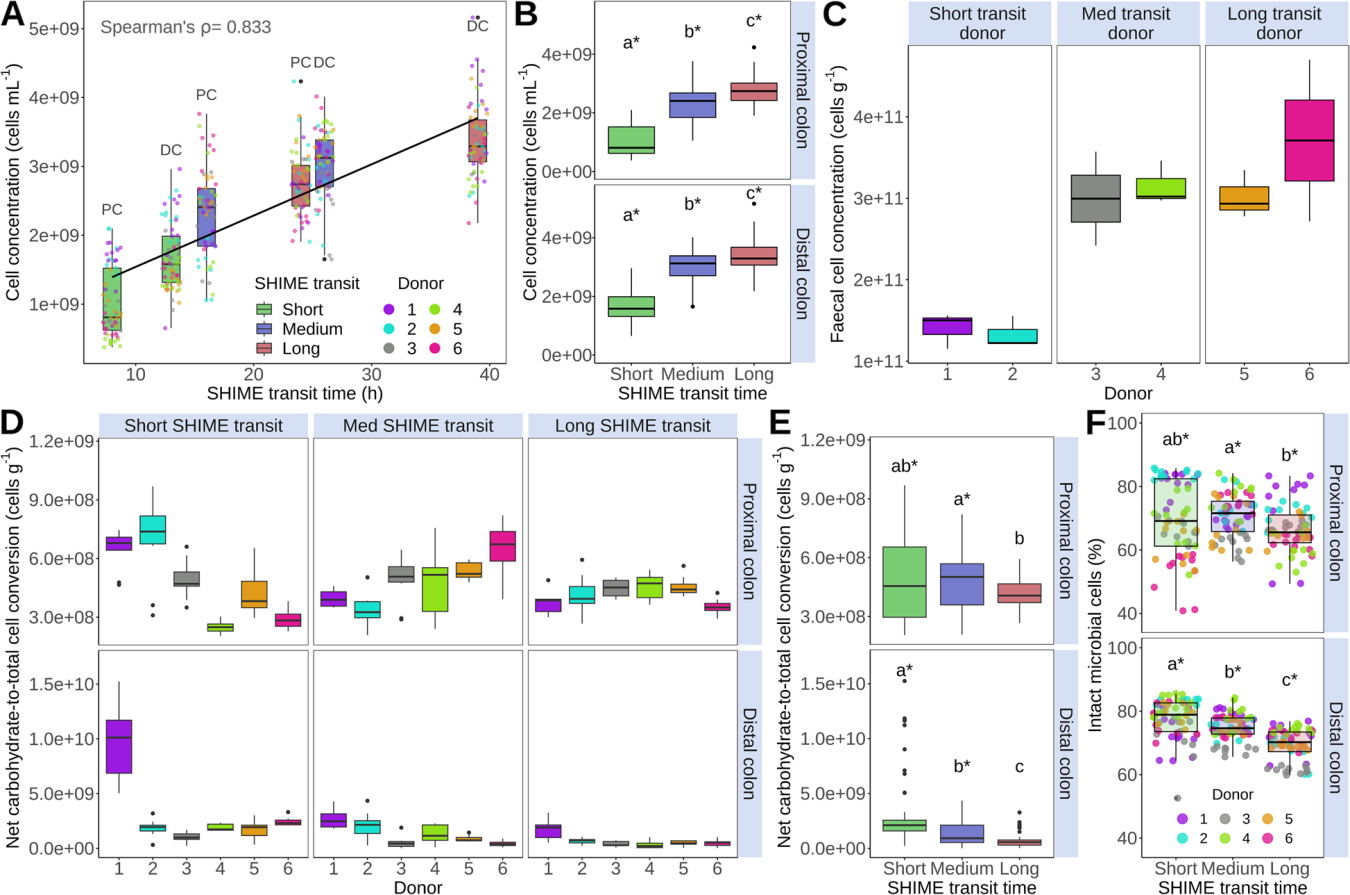
**A** Cell concentrations (cells mL^−1^) and SHIME transit time (proximal colon (PC) = 8, 16 and 24h; distal colon (DC) = 13, 26 and 39h) significantly correlated (*n* = 60, *P* = 4E-99, Spearman’s rank correlation). **B** Total cell concentrations (cells mL^−1^) significantly increased with SHIME transit time (*n* = 60). **C** The faecal cell concentrations (cells g^−1^) from the six donors increased with their self-assessed corn in vivo transit time (*n* = 12, Table S1). **D**–**E** The carbohydrate-to-total cell conversion (cells g^−1^) significantly decreased with SHIME transit time (*n* = 15 per donor, *P* = 1.35E-12). **F** The percentage of intact microbial cells (%) decreased with SHIME transit time (*n* = 60). Statistically significant differences between SHIME transit times are indicated by the letters a, b and c in panels **B**, **E** and **F** (unpaired two-sided Wilcoxon signed rank tests with Holm correction). Identical letters indicate no significant differences (*P* > 0.05). Significant differences between colon regions are indicated with asterisks (*) (*P* < 0.05, paired two-sided Wilcoxon signed rank tests with Holm correction). Box plots display the interquartile range, median and outliers beyond the 1.5 times interquartile range (whiskers). In A and F, individual data points are added

Transit time, with an explanatory power of 52%, was the main driver of microbial cell concentrations in the SHIME, outweighing inter-individual variability accounting for 2.7% of the variation in microbial load (*P*_adjusted_ = 0.004, Figure S6). The microbial load significantly increased 2.7-fold in the proximal and twofold in the distal colon at long compared to short transit time (Fig. 1A–B, Spearman’s *ρ* = 0.833, *P* = 4.00E-99). This significant in vitro correlation is in line with the increasing trend in faecal microbial cell counts per gram wet weight observed in vivo in long transit donors (Fig. 1C). Higher cell concentrations in slower transits coincided with a significantly higher net carbohydrate utilisation, which increased 3.1-fold (*P* = 3.21E-07) in the proximal and 3.6-fold (*P* = 1.09E-07) in the distal colon, at long compared to short transit time (Figure S7). In contrast, the biomass production efficiency, i.e. the net-carbohydrate-to-total cell conversion, significantly decreased with decelerated transit time in the distal colon (*P* = 1.35E-12, Fig. 1D–E). The enhanced carbohydrate utilisation efficiency at short transit time is a necessary adaptation of the resident microbiota to avoid washout. The adaptive capacity to grow under short transit SHIME conditions was higher in faecal microbiomes from donors with short in vivo transit. This was reflected in a 2.2-fold higher cell density and an almost twofold higher net carbohydrate-to-biomass conversion of faecal microbiomes derived from donors with short (donor 1–2) versus longer (donor 3–6) in vivo transit in the proximal short transit SHIME compartment (Fig. 1D–E). Intact cell growth per gram of utilised carbohydrates was even 2.5-fold higher in short in vivo transit donors (Figure S8). Consequently, the proportion of intact cells depended more on inter-individual differences (*R*^2^_adjusted_ = 0.15) and in vivo transit time (*R*^2^_adjusted_ = 0.09) than on SHIME transit time variation (*R*^2^_adjusted_ = 0.08, *P*_adjusted_ = 0.004, Figure S6). Nevertheless, the intact cell ratio significantly differed between medium and long SHIME transit times in the proximal colon (*P* = 0.001, Fig. 1F). In the distal colon, intact cell percentages significantly decreased (*P* = 0.013, Fig. 1F).

### SHIME transit time variation significantly affected the proportional and quantitative microbial community composition

**Fig. 2:**
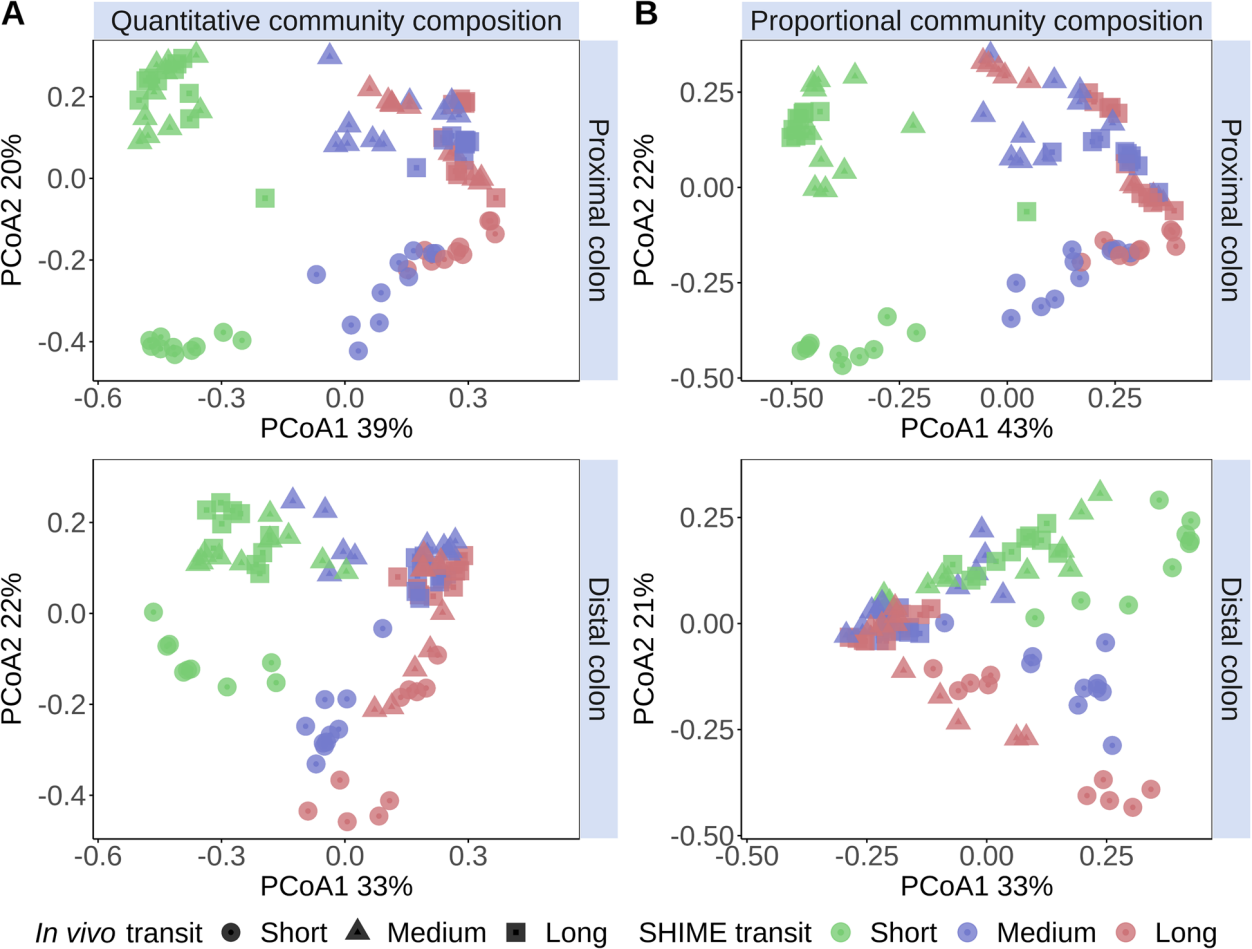
Principal coordinates analysis (PCoA, *n* = 90) of the genus-level quantitative microbial community compositions (**A**) and proportional microbial community compositions (**B**), based on Bray–Curtis dissimilarity, demonstrates a clustering by SHIME transit time (colour) along the first PCoA dimension and by the self-assessed in vivo corn transit time (shape, Table S1) along the second PCoA dimension. Short, medium and long SHIME transit times were 8, 16 and 24h in the proximal colon and 13, 26 and 39h in the distal colon

Transit time was the main driver of the proportional (PMP) and quantitative microbial profiles (QMP). An unsupervised ordination revealed a clustering of the QMP and PMP according to SHIME transit time along the first PCoA dimension (Fig. 2). In line with this, SHIME transit time explained 24% of the variation in QMP and 22% of the variation in PMP at genus level (*P*_adjusted_ = 0.004, Figure S9). Interindividual differences explained 22% in QMP and PMP variation in genus level microbial community composition (*P*_adjusted_ = 0.004, Figure S9). In vivo transit time accounted for 14% of QMP and 13% of PMP variation (*P*_adjusted_ = 0.004, Figure S9) which corresponded with a separate clustering of short versus medium and long in vivo transit time donors along the second PCoA dimension (Fig. 2).

### The microbiome response to transit time is species-specific and consistent across faecal donors

**Fig. 3:**
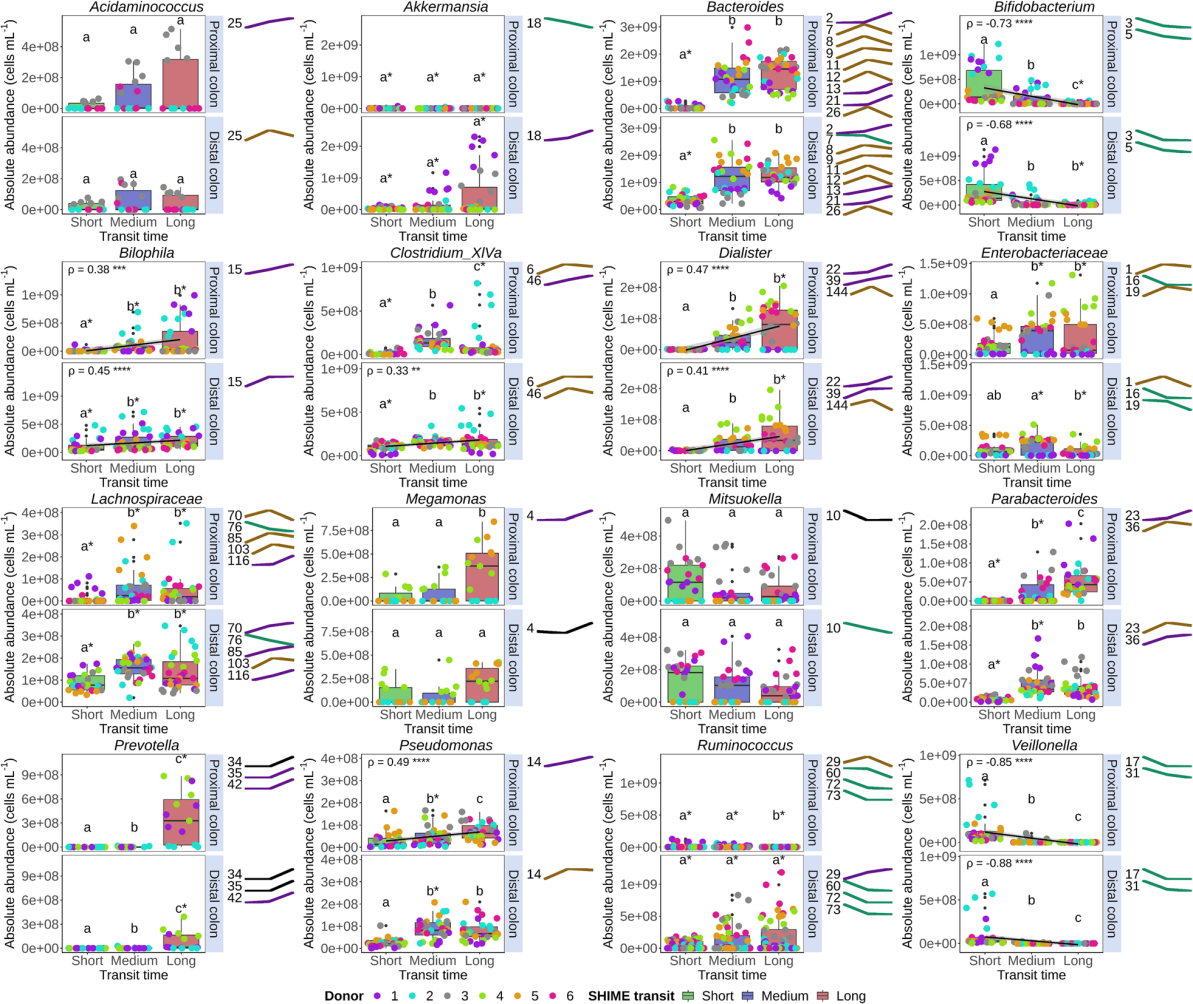
Absolute abundance (cells mL.^−1^) of the most abundant genera and species (represented as OTU trend lines) varied with SHIME transit time in the proximal and distal colon region (*n* = 30). Only significant OTUs with a relative abundance of more than 5% within the genus were shown. Short, medium and long SHIME transit times were 8, 16 and 24h in the proximal colon and 13, 26 and 39h in the distal colon. Statistically significant differences between SHIME transit times are indicated by the letters a, b and c (unpaired two-sided Wilcoxon signed rank tests with Holm correction). Identical letters indicate no significant differences (*P*_FDR_ > 0.05). Significant differences between colon regions are indicated with asterisks (*) (*P*_FDR_ < 0.05, paired two-sided Wilcoxon signed rank tests with Holm correction). Spearman’s rank correlation coefficients (*ρ*) and corresponding P_FDR_-values (*P_FDR_ < 0.05, **P_FDR_ < 0.01, ***P_FDR_ < 0.001, ****P_FDR_ < 0.0001) were only calculated for monotonic relationships including only the donors carrying the taxon of interest. Higher level taxa are to be interpreted as unclassified genus belonging to the respective taxon. Box plots display individual data points, as well as the interquartile range, median and outliers beyond the 1.5 times interquartile range (whiskers)

**Fig. 4:**
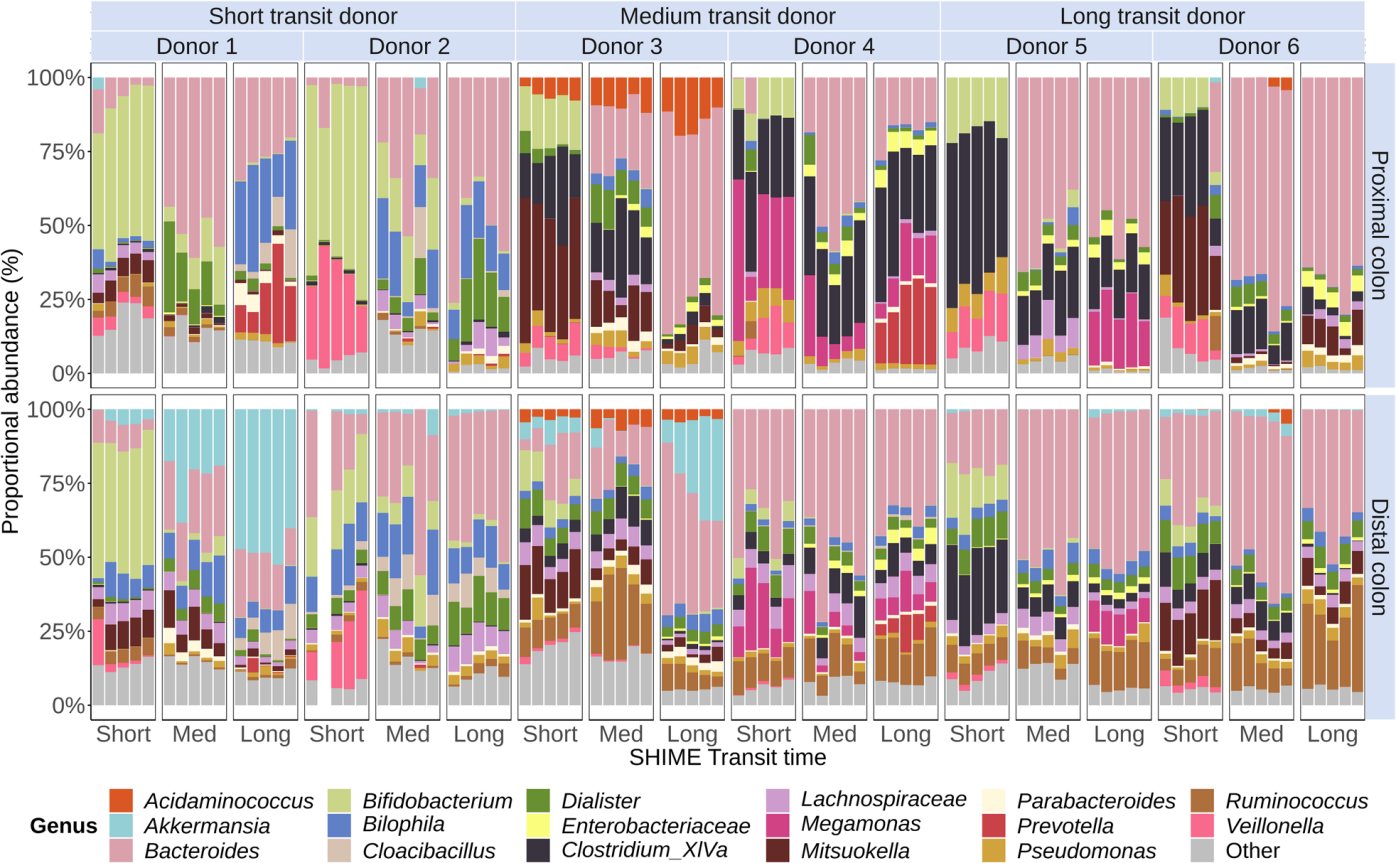
Proportional abundance (%) of the 17 most abundant genera varied with SHIME transit time in the proximal and distal colon region simulating six donors with short, medium and long self-assessed in vivo corn transit time (Table S1). Short, medium and long SHIME transit times were 8, 16 and 24h in the proximal colon and 13, 26 and 39h in the distal colon. Less abundant genera are binned into ‘Other’. Higher level taxa are to be interpreted as the unclassified genus belonging to the respective taxon

Grouping all donors (*n* = 6), 13 out of the 17 most abundant genera were significantly affected in relative and absolute abundance by transit time variation. Most genera displayed significant positive correlations with transit time. *Bacteroides* absolute abundances significantly increased 20.2-fold in the proximal (*P*_FDR_ = 8.05E-17) and 3.6-fold in the distal (*P*_FDR_ = 9.71E-12) region of the long compared to the short transit SHIME (Figs. 3 and S10). Proportional differences were smaller but still significant (Fig. 4). Decomposition of the increasing *Bacteroides* abundances at species level revealed increases in OTU2 (*Phocaeicola vulgatus*), OTU9 (*Phocaeicola massiliensis*) and OTU13 (*Bacteroides uniformis*) (Fig. 3). *Parabacteroides* OTU26 (*P. distasonis*) and OTU36 (*P. merdae*), closely related former *Bacteroides* species, also showed significant positive correlations with transit time. OTU12 (*B. caccae*), on the other hand, remained unaffected, except in donor 6 displaying a peak in the medium transit time. OTU8 (*B. thetaiotaomicron*), OTU11 (*B. fragilis*) and OTU7 (*B. kribbi*) also peaked in the medium SHIME transit proximal compartment. OTU7 significantly decreased at longer transit times in the distal colon (Figs. 3 and 4).

While *Bacteroides* OTUs exhibited a diverging response to transit time variation, *Prevotella* OTUs, just like *Bacteroides* predominantly associated with high-fibre diets [42, 43], consistently portrayed significantly higher abundances at long transits (*P*_FDR_ < 0.0001, Fig. 3). *Ruminococcus*, another primary degrader of complex fibres [44], had significantly lower absolute (*P*_FDR_ = 1.36E-05) and proportional (*P*_FDR_ = 1.72E-05) abundances in longer transits in the proximal colon. *Ruminococcus* OTU60 (*Mediteraneibacter faecis*), OTU72 (*R. lactaris*) and OTU73 followed the genus-level trends in the proximal colon. In contrast, OTU29 (*Ruminococcus torques*) significantly increased in absolute (*P*_FDR_ = 3.03E-05) and proportional (*P*_FDR_ = 4.14E-03) distal colon abundances with longer SHIME transit (Figs. 3 and S10).

Overall, genera that rely on the fermentation of less complex carbohydrates displayed a lower relative and absolute abundance with longer SHIME transit times. *Bifidobacterium* represented by OTU3 (*B. adolescentis*) and OTU5 (*B. pseudocatenulatum*) significantly decreased due to a longer SHIME transit in the proximal (*P*_FDR_ = 1.51E-15) and distal colon (*P*_FDR_ = 2.41E-12). Likewise, *Veillonella* OTU17 (*V. dispar*) and OTU31 (*V. rogosae*), *Mitsuokella* OTU10 (*M. jalaludinii*) and unclassified *Enterobacteriaceae* OTU16 and OTU19 (in the distal colon) were less abundant at longer SHIME transit times (Figs. 3 and S10). In contrast, other less complex carbohydrate fermenting *Dialister* OTU22 (*D. hominis*) and OTU39 (*D. invisus*) and *Megamonas* OTU4 (*M. funiformis*) increased with transit time (Fig. 3).

Nutrient-niche-specific bacteria, such as *Bilophila* (OTU15, *B. wadsworthia*) and *Akkermansia* (OTU18, *A. muciniphila*, only present in high abundances in donors 1 and 3), reached the highest proportional and absolute abundances at long SHIME transit (Figs. 3 and 4). Similarly, amino-acid-degrading bacteria *Cloacibacillus* (OTU38, *C. evryensis*), *Pseudomonas* (OTU14, *P. aeruginosa*) and *Acidaminococcus* (OTU25, *A. intestini*) increased with transit time (Figs. 3 and S10).

Butyrate producing genera *Blautia* (OTU40, *B. wexlerae* and OTU74, *B. luti*) and *Clostridium cluster XIVa* (OTU6, *Enterocloster bolteae*) significantly peaked in absolute and proportional abundances in the medium SHIME transit time (*P*_FDR_ < 0.0001), except for the proportional abundances in the distal colon (*P*_FDR_ = 0.34, *P*_FDR_ = 0.10, Figs. 3 and S10). Likewise, OTU30 (*Eubacterium rectale*) and OTU61 (*Anaerobutyricum hallii*), belonging to the *Clostridiales* order showed the highest absolute and proportional abundances in the medium transit SHIME (Figure S10). Unclassified *Lachnospiraceae* OTU70, OTU85 and OTU103, a family often linked with butyrate production [45], also portrayed the highest absolute and proportional abundances in the medium SHIME transit (Fig. 3), while the absolute (*P*_FDRproximal_ = 3.3E-04 and *P*_FDRdistal_ = 1.2E-03) and proportional (*P*_FDRproximal_ = 3.6E-04 and *P*_FDRdistal_ = 1.0E-03) abundances of the butyrate producing *Faecalibacterium* (OTU24 and OTU47, *F. prausnitzii*) significantly dropped in medium and long SHIME transit compared to the short transit time (Figure S10).

### Decelerating SHIME transit increased net short-chain fatty acid production but the carbohydrate-to-SCFA conversion efficiency varied with donor, colon region and SCFA chain length

**Fig. 5:**
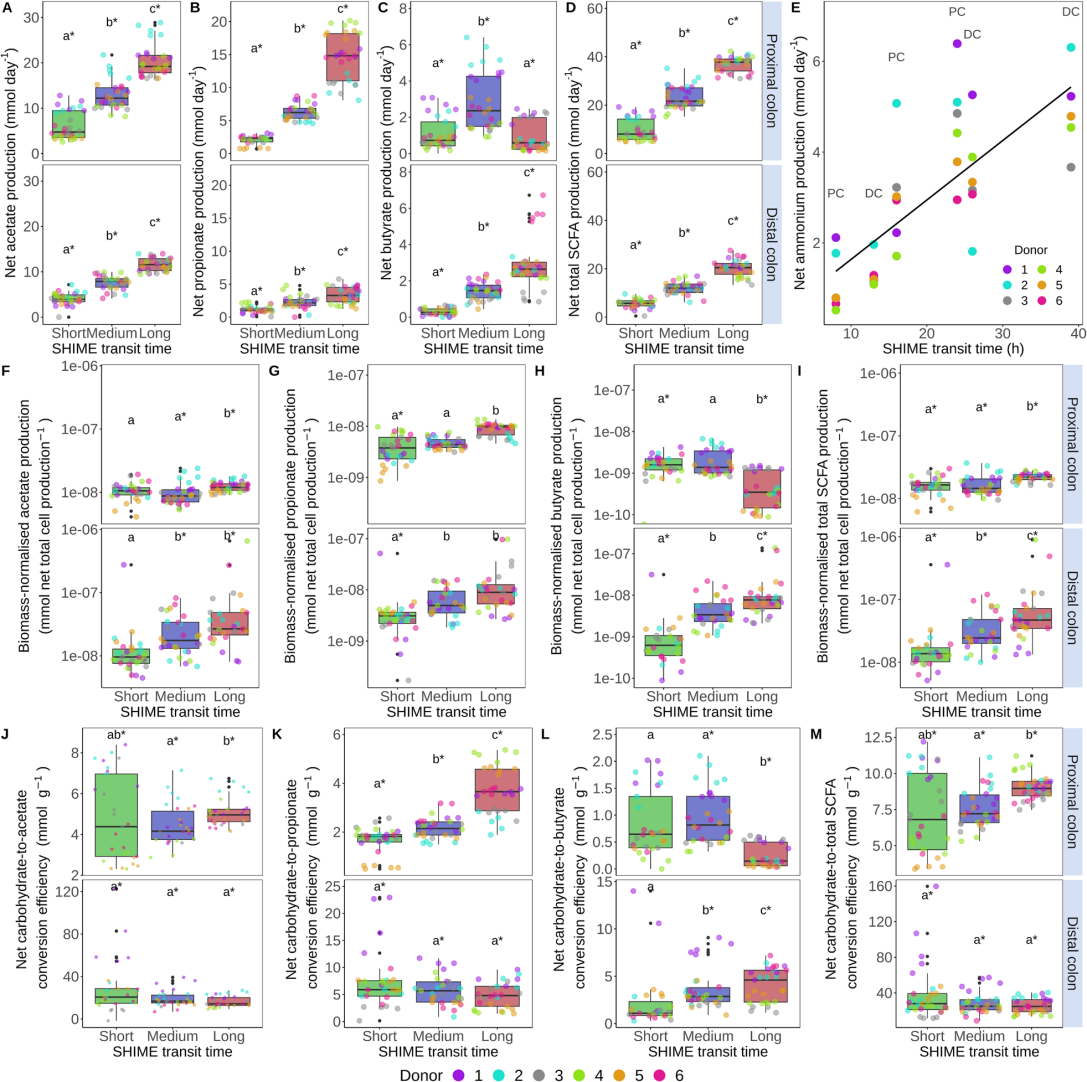
Net daily production (mmol day^−1^) of acetate (**A**), propionate (**B**), butyrate (**C**) and total SCFA (**D**) was significantly affected by SHIME transit time variation (*n* = 30). (**E**) Net daily ammonium production (mmol day^−1^) and SHIME transit time significantly correlated (*n* = 12, *P* = 1.08E-05, Spearman’s rank correlation). The biomass-normalised net acetate (**F**), propionate (**G**), butyrate (**H**) and total SCFA (**I**) production relative to the net biomass production (mmol total cells^−1^) significantly changed with SHIME transit time (*n* = 30). The net carbohydrate-to-acetate (**J**), propionate (**K**), butyrate (**L**) and total SCFA (**M**) conversion efficiency, i.e. the net production relative to the daily carbohydrate utilisation (mmol g.^−1^), was significantly affected by SHIME transit time (*n* = 30). Short, medium and long SHIME transit times were 8, 16 and 24h in the proximal colon and 13, 26 and 39h in the distal colon. Statistically significant differences between transit times are depicted by the letters a, b and c in panels **A**–**D** and **F**–**M** (unpaired two-sided Wilcoxon signed rank tests with Holm correction). Identical letters indicate no significant differences (*P* > 0.05). Significant differences between colon regions of the same transit time are marked with asterisks ( ∗) (*P* < 0.05, paired two-sided Wilcoxon signed rank tests with Holm correction). Box plots display individual data points, as well as the interquartile range, median and outliers beyond the 1.5 times interquartile range (whiskers)

Transit time was the main driver of the daily net metabolite production (*P*_adjusted_ = 0.004, *R*^2^_adjusted_ = 0.45, Figure S11). An increased transit time in the SHIME led to a significantly higher net acetate, propionate and total SCFA production (Fig. 5A, B, D). The net daily production rates peaked in the long transit at 20.31 ± 3.61mmol acetate day^−1^ in the proximal and 11.63 ± 1.43mmol acetate day^−1^ in the distal colon and 14.64 ± 3.65mmol propionate day^−1^ in the proximal and 3.36 ± 1.22mmol propionate day^−1^ in the distal colon (Fig. 5A–B). The highest daily net butyrate production was observed at medium transit time in the proximal (2.84 ± 1.62mmol day^−1^) and at long transit time in the distal colon (2.92 ± 1.46mmol day^−1^, Fig. 5C). Integrative analysis revealed positive correlations between the net daily butyrate production and the absolute abundances of *Anaeroglobus* (*r* = 0.69), *Blautia* (*r* = 0.77), unclassified *Clostridiales* (*r* = 0.71) and *Roseburia* (*r* = 0.55, Figure S12). Net daily ammonium production, a marker for proteolytic activity, showed a positive correlation with transit time (*ρ* = 0.662, *P* = 1.08E-05, Fig. 5E).

Normalising the net SCFA production relative to the net biomass production levelled out the largest differences between transit times in the proximal colon, with the exception of the butyrate production, which was 5.98E-10 ± 5.25E-10mmol cells^−1^ lower in the long transit (*P* = 8.8E-13). Significant increases in the biomass-normalised SCFA production as a function of decelerated transit were still observed in the distal colon and for acetate and propionate in the proximal colon (Fig. 5F–I). Thus, microbial cells produced SCFA more efficiently due to a slower transit (Fig. 5I). The increased efficiency reflected an increased carbohydrate fermentation at longer transit times in the proximal colon (Figure S7).

The carbohydrate-to-propionate conversion efficiency significantly increased with transit time in the proximal colon. The carbohydrate fermentation to butyrate was significantly less efficient in the proximal and significantly more efficient in the distal colon at longer SHIME transit times. The carbohydrate-to-acetate conversion efficiency was invariable (Fig. 5J–M). Besides colon region and SCFA chain length, the metabolite production efficiency in terms of the carbohydrate utilisation depended on the donor. The total SCFA production efficiency in the proximal colon decreased from short to long SHIME transits in donors 1 and 2 with short and donor 3 with medium in vivo transit time. Conversely, medium in vivo donor 4 and long in vivo donors 5 and 6 displayed increasing carbohydrate conversion efficiency as SHIME transit time increased. This peak in efficiency at in vitro transit times akin to the in vivo transit time of the donor suggests an adaptation of the human microbiome to transit time which hence acts as an important driver of inter-individual variability (Fig. 5M).

The branched SCFA, isovalerate and isobutyrate, followed the trends in butyrate production (Figure S13F, G, K, L). Positive correlations were obtained between isobutyrate production and *Anaeroglobus* (*r* = 0.57), *Blautia* (*r* = 0.56) and *Clostridiales* (*r* = 0.62) absolute abundances. *Blautia* also positively correlated with the isovalerate production (*r* = 0.59, Figure S12).

## Discussion

Transit time is the main determinant of the variation in microbial cell counts (*R*^2^_adjusted_ = 52%), absolute and proportional gut microbial community composition (*R*^2^_adjusted_ = 24 and 22%) and metabolic activity (*R*^2^_adjusted_ = 45%) in the in vitro SHIME. Transit time has already been reported to affect the quantitative (*R*^2^_adjusted_ = 4.3%) and proportional community compositions (*R*^2^_adjusted_ = 7.3%) in vivo [8]*.* However, in vivo effect sizes are more limited due to confounding factors, including diet, obscuring the link between transit time and human faecal microbiome variation [7, 23]. Our unique approach to disentangle transit time from other confounding factors in the absence of the host physiological complexity allowed us to identify some spurious in vivo correlations.

Our finding that *Prevotella* exclusively thrives at long transit times is in line with its reported slow growth and slow complex fibre degradation [46], but contests the previously established in vivo link between the *Prevotella* enterotype and loose stools, which are indicative of a short transit time [3]. The in vivo link is confounded by diet since *Prevotella* is typically associated with a high non-fermentable fibre intake, which is suggested to increase the stool water holding capacity and exert a faecal bulking effect that accelerates transit [43, 47]. We thus refute the hypothesis that a rapid gut transit drives the *Prevotella*-enterotype [3].

*Ruminococcus*, another genus of complex fibre degraders [42–44, 48], portrayed higher absolute and proportional abundances at shorter transits in the SHIME, which contrasts the positive in vivo correlation between *Ruminococcaceae*, firm stools and long transit times. This discrepancy is likely attributed to the observed species-specific response to transit time variation which may be prompted by the diverging and selective substrate preferences of different *Ruminococcus* species [49]. *Ruminococcus lactaris*, a species that can utilise resistant starch grew better at a shorter transit than mucinolytic *R. torques* [50, 51]*. R. torques* harbours only a small fraction (fucoses and galactosidases in GH families 2,29,95) of the extensive (carbohydrate active) enzyme complement required to break down structurally complex mucins [51]. As a consequence, *R. torques* enrichment in the long transit SHIME could be due to its dependency on cross-feeding with other mucus-degrading species that express sialidases (GH33), N-acetyl-glucosaminidases (GH84, GH85, G89, GH20) and N-acetyl-galactosaminidases (GH101, GH129) such as *Akkermansia muciniphila* which is positively correlated with transit time in vivo [3, 28]*.*

*Akkermansia* indeed reached the highest proportional and absolute abundances at long SHIME transit time, as did bile-tolerant *Bilophila*. Higher abundances of *Bilophila* and *Akkermansia* have consistently been linked with nutrient depletion which is more pronounced at longer transit times as apparent from the increased net carbohydrate consumption in the long transit SHIME [3, 28]. The bloom of mucus degraders in vitro, furthermore, coincides with the increased in vivo mucus degradation with prolonged transit [52]. The depletion of easily fermentable carbohydrates at longer transit times is also known to induce proteolysis and amino acid fermentation [9, 53–57], explaining the surge we observed in amino-acid-degrading *Cloacibacillus* and *Acidaminococcus* in the long transit SHIME and the positive correlation of the net daily ammonium production, a marker for proteolytic activity, with transit time. Increased proteolytic activity, including faecal ammonium levels, were also reported in vivo in individuals with a delayed transit [11, 52, 58, 59]. Protein fermentation products and the aforementioned erosion of the protective mucus layer have potentially detrimental health effects. Transit time, with fibre deprivation as an underlying factor, could, therefore, be implicated in a number of gut microbiota-related diseases such as colon cancer and diverticulosis coli [9, 60].

Fibre deprivation occurs when the rate of carbohydrate utilisation by the microbiota exceeds the fibre intake rate. The carbohydrate metabolism rate depends on the microbial population density, which is significantly higher at a prolonged SHIME and in vivo transit [6]. Our in vitro approach, however, also revealed a decreased carbohydrate-to-biomass conversion efficiency, indicating that a slower transit in the system did not increase growth rates but instead resulted in biomass accumulation [61, 62]. On the contrary, a slower transit induced lower growth rates in chemostats and correlated with a lower faecal microbiota growth potential in vivo, which is consistent with a lower selective pressure [3, 63].

In silico predicted anaerobic growth rates, derived from the Assembly of Gut Organisms through Reconstruction and Analysis (AGORA) models [64], confirmed a shift from faster to slower growing species with increasing transit time. The nutrient-degrading specialists such as *Akkermansia*, *Bilophila*, *Cloacibacillus*, *Acidaminococcus*, *R. torques* and *Prevotella* (0.084 ± 0.032h^−1^) have slow predicted growth rates compared to *Bifidobacterium* and *Veillonella* (0.170 ± 0.074h^−1^). *Bifidobacterium* has a broad fermentation capability, ranging from simple sugars to complex carbohydrates such as pectin, mucin and oligosaccharides [65–68]. This high substrate versatility could be advantageous at high substrate passage rate characteristic for faster transits. *Veillonella* enrichment could be favoured by its capacity to utilise lactate, an end product of carbohydrate fermentation produced by, amongst others, *Bifidobacterium* species [67, 69]. *Bacteroides* spp., despite being glycan degrading specialists with a rapid growth (0.419 ± 0.159h^−1^), were enriched at longer transit times in vitro and in vivo [4]*.* This indicates that, besides growth rate, other factors such as substrate affinity, pH or in vivo transit time of the faecal microbiome donor dictate the response to transit time variation.

Donor in vivo transit time explained more than 10% of the microbiota variation in the SHIME and faecal microbiomes derived from short in vivo transit time donors reached approximately twofold higher cell densities and carbohydrate-to-biomass conversion efficiencies in the SHIME compared to microbiota obtained from longer in vivo transit time donors. This microbiota adaptation to in vivo transit time is further underscored by our observation that the carbohydrate-to-SCFA conversion was most efficient at in vitro transit times similar to the in vivo transit time of the donor microbiota. When all donors were grouped, net carbohydrate-to-SCFA conversion and total SCFA production increased with transit time, in agreement with previous in vitro studies [70]. This contrasts with reduced in vivo faecal SCFA concentrations but aligns with increased in vivo SCFA concentrations in the ascending colon of sudden death victims with longer transits [7, 61, 62, 71, 72]. Faecal SCFA concentrations are thus lowered despite an elevated SCFA production, due to an increased gastrointestinal SCFA absorption at a longer residence time. Differential absorption of SCFA that vary in chain length can also distort the relative SCFA profiles measured in faecal samples. Our SHIME study, in contrast, provides an unbiased view on the shifting SCFA ratios with transit time.

An increased propionate production coincided with enrichment of slow growing propionate producing *Prevotella*, *Akkermansia*, *Bacteroides uniformis*, *Phocaeicola vulgatus*, *Dialister invisus* and *Phascolarctobacterium faecium* at a prolonged transit time [73–75]. The AGORA-predicted growth rates across propionate producers are slower (0.088 ± 0.058h^−1^) than in butyrate producers (0.239 ± 0.100h^−1^), except for *P. vulgatus* (0.520h^−1^) [64]. In line with this, the proximal net butyrate production significantly decreased in the long SHIME transit time after an initial increase from short to medium transit. This initial shift was positively correlated with the absolute abundances of the butyrate producing genera *Anaeroglobus*, *Blautia*, *Clostridiales* and *Roseburia*. *Roseburia* has been reported in lower in vivo abundances at longer transit times [4]. *Clostridium cluster XIVa* and *Faecalibacterium* followed a similar trend, however, statistically non-significant. The difference in growth performance between propionate and butyrate producers also resulted in a shift from a significant positive correlation between propionate and butyrate production in the short transit towards a significant negative correlation in the long transit (Figure S14). Transit time thus clearly impacted microbial metabolism, next to the microbiota composition, underlining its importance for future microbiome research.

Adjustment of in vitro transit time based on in vivo data has never been performed prior to this study. Most SHIME experiments previously applied colonic transit times of either 52h in a two-stage or 76h in three-stage colon setup [13, 28, 76, 77]. Standardised transit times are common practice in in vitro research. For example, the artificial colon model (ARCOL), Polyfermentor Intestinal Model (PolyFerm-S) and SIMulator GastroIntestinal (SIMGI) have static transit times of 72, 7.5 and 76h, respectively [78–80]. The TNO in vitro model (TIM-2) and Dynamic Colon Model (DCM), both tubular systems mimicking the in vivo peristaltic propulsion, are also operated in a standardised manner [81–83]. This pragmatic standardised in vitro approach has yielded valuable mechanistic insights in microbiome dynamics and response but it may obscure and even distort the interindividual variability in response to gut microbiome determinants. *Veillonella*, for instance, remained undetected in the long transit SHIME, whereas *Prevotella* was not observed at the short transit SHIME runs. A standardised model would have overlooked both genera, confounding the in vitro analysis.

## Conclusions

We propose an in vitro approach with transit time personalisation as a novel powerful tool to improve the fundamental ecological insights into the human gut microbiome. Transit time personalisation is, moreover, a stepping stone to personalised in vitro research which is essential to more accurately predict an individual’s microbiome response to (dietary) interventions. Such accurate prediction allows for a more targeted personalised treatment that fits well in the personalised medicine framework that will lower the burden on global healthcare systems by decreasing healthcare costs due to the elimination of trial and error therapies. Amongst others, transit time modification with diet and pre- and probiotic supplementations could be explored as a therapeutic strategy to shift the microbiome towards a healthy state since transit time aberrancies have been linked with microbiome-mediated diseases. Even when transit time is not the primary focus, we advocate the measurement of transit time as a confounding factor in clinical trials and in observational studies aiming to understand the interindividual microbiome variability across health status gradients.

### Supplementary Information

**Additional file 1.**

## Data Availability

Sequencing data have been deposited in the European Bioinformatics Institute’s (EBI) European Nucleotide Archive (ENA) with the accession code ERP138715. Flow cytometry data can be accessed in the FlowRepository archive with the accession code FR-FCM-Z5K7. All code used for statistical analysis is available in GitHub (https://github.com/yminnebo/TransitSHIME).

## Abbreviations

AGORA: Assembly of Gut Organisms through Reconstruction and Analysis
ARCOL: Artificial colon model
BSS: Bristol Stool Scale
db-RDA: Distance-based redundancy analysis
DCM: Dynamic Colon Model
EBI: European Bioinformatics Institute
ENA: European Nucleotide Archive
LT: Long Transit
MT: Medium Transit
OTU: Operational Taxonomic Unit
PCoA: Principal Coordinates Analysis
PMP: Proportional Microbial Pofiles
PolyFerm-S: Polyfermentor intestinal model
QMP: Quantitative Microbial Profiles
SCFA: Short-Chain Fatty Acids
SHIME: Simulator of the Human Intestinal Microbial Ecosystem
SIMGI: SIMulator GastroIntestinal
sPLS: sparse Partial Least Squares
ST: Short Transit
TIM-2: TNO in vitro model
