# Supplementary material for: Gut microbiota response to in vitro transit time variation is mediated by microbial growth rates, nutrient use efficiency and adaptation to in vivo transit time
Source: Microbiome. 2023 Nov 6;11:240. doi: 10.1186/s40168-023-01691-y (PMC10626715; doi:10.1186/s40168-023-01691-y)
Supplement: Supplementary file 2 — Additional file 1. [file 40168_2023_1691_MOESM1_ESM.docx]

# Supplementary Note

## Note S1: Correlation between Bristol Stool Scale (BSS) and transit time

Measurement of transit time can be performed with advanced magnetised capsules, dyes, scintillated markers, but relatively simple and accessible parameters such as the Bristol Stool Scale (BSS), stool frequency and corn passage time, also produced a consistent and highly correlated estimate of transit time (Figure S15) [1–7].

While stool consistency is significantly negatively correlated with whole-gut transit time, correlation coefficients ranging from -0.54 to −0.84 imply that other factors besides transit time affect the BSS [3,8,9]. One such factor is bias introduced by self-assessment of the BSS [10]. This type of observer-bias was eliminated *in vitro* since transit time can be accurately and directly modified by adjusting the SHIME flow rate and volume. The BSS of the faecal samples used to inoculate the SHIME was, moreover, determined by a single investigator and validated by the independent measurement of the self-reported corn transit time and stool frequency to rule out observer-bias during the selection of donors based on *in vivo* transit times. The relationship between the easily self-assessable stool defecation frequency and transit time is still under debate [2,8,9]. However, our results, with a limited sample size, indicate strong correlations between stool frequency and BSS (ρ=0.912, P=0.01, Figure S15A), and frequency and *in vivo* corn transit time (ρ=0.955, P=0.003, Figure S15B) [2]. The correlation between the *in vivo* corn transit time and BSS was also stronger (ρ=-0.9, P=1.77E-06, n=6, Figure S15C) than previously reported by Nestel et al (2021) (ρ=-0.37, P=0.049, n=25) [11]. This relates to the fact that the BSS represents the combined effect of transit time and faecal water holding capacity. A longer residence in the colon, i.e. longer colonic transit time, favours a higher degree of water absorption, resulting in firmer stools and lower BSS. However, certain fibre sources with a high water-holding capacity may result in looser stools and increased BSS, even at longer transit times [12]. Particularly because the water-holding capacity and faecal bulking effects of fibres have been shown to be independent [12–14]. Nevertheless, water absorption and transit time are intrinsically linked *in vivo* but absorption was not simulated *in vitro*.

The lack of a water absorption step in our SHIME model does not interfere with our results since *in vivo* water absorption does not lower the water activity of faecal samples (and thus gut contents) below a value of 0.97, implying that microbial growth is not affected by water stress and microbial enzymatic activity is not reduced. Water activity was, moreover, unrelated to moisture content or BSS, confirming that the effects of transit time on the gut microbiome are not mediated by differences in water activity [15,16]. In fact, another consequence of omitting absorption *in vitro* is that the measured SCFA concentrations directly reflect SCFA production whereas faecal SCFA concentrations are the result of production, cross-feeding and gastrointestinal (SCFA and water) absorption, which is influenced by, amongst others, diet, intestinal bulk and pharmaceuticals [17]. Faecal SCFA concentrations can, therefore, be misleading. This is exemplified by the fact that a longer transit corresponds with decreased faecal SCFA concentrations in *vivo*, but increased the total SCFA, acetate and propionate production in our SHIME [18–20]. Our *in vitro* results are in line with increased SCFA concentrations observed in the ascending colon of sudden death victims with longer transits [21,22]. Given the inaccessibility of different gut regions *in vivo*, however, an *in vitro* approach is most appropriate to disentangle transit time from confounding factors as well as proxy variables, such as stool consistency estimated through the Bristol Stool Scale (BSS) and faecal moisture content, which are often used as an indicative, indirect measure for transit time [1–7].

**References Supplementary note**

[1] D. Vandeputte, G. Falony, S. Vieira-Silva, R. Y. Tito, M. Joossens, and J. Raes, ‘Stool consistency is strongly associated with gut microbiota richness and composition, enterotypes and bacterial growth rates’, *Gut*, vol. 65, no. 1, pp. 57–62, Jan. 2016, doi: 10.1136/gutjnl-2015-309618.

[2] F. Asnicar *et al.*, ‘Blue poo: impact of gut transit time on the gut microbiome using a novel marker’, *Gut*, vol. 70, no. 9, pp. 1665–1674, Sep. 2021, doi: 10.1136/gutjnl-2020-323877.

[3] S. J. Lewis and K. W. Heaton, ‘Stool form scale as a useful guide to intestinal transit time’, *Scandinavian Journal of Gastroenterology*, vol. 32, no. 9, pp. 920–924, Jan. 1997, doi: 10.3109/00365529709011203.

[4] D. Vandeputte *et al.*, ‘Temporal variability in quantitative human gut microbiome profiles and implications for clinical research’, *Nat Commun*, vol. 12, no. 1, Art. no. 1, Nov. 2021, doi: 10.1038/s41467-021-27098-7.

[5] M. S. Cirstea *et al.*, ‘Microbiota Composition and Metabolism Are Associated With Gut Function in Parkinson’s Disease’, *Movement Disorders*, vol. 35, no. 7, pp. 1208–1217, 2020, doi: 10.1002/mds.28052.

[6] L. P. Degen and S. F. Phillips, ‘Variability of gastrointestinal transit in healthy women and men’, *Gut*, vol. 39, no. 2, pp. 299–305, Aug. 1996, doi: 10.1136/gut.39.2.299.

[7] M. Valles-Colomer *et al.*, ‘Variation and transmission of the human gut microbiota across multiple familial generations’, *Nature Microbiology 2021 7:1*, vol. 7, no. 1, pp. 87–96, Dec. 2021, doi: 10.1038/s41564-021-01021-8.

[8] R. J. Saad *et al.*, ‘Do stool form and frequency correlate with whole-gut and colonic transit results from a multicenter study in constipated individuals and healthy controls’, *American Journal of Gastroenterology*, vol. 105, no. 2, pp. 403–411, Feb. 2010, doi: 10.1038/AJG.2009.612.

[9] M. Russo *et al.*, ‘Stool consistency, but not frequency, correlates with total gastrointestinal transit time in children’, *The Journal of Pediatrics*, vol. 162, no. 6, pp. 1188–1192, Jun. 2013, doi: 10.1016/J.JPEDS.2012.11.082.

[10] M. R. Blake, J. M. Raker, and K. Whelan, ‘Validity and reliability of the Bristol Stool Form Scale in healthy adults and patients with diarrhoea-predominant irritable bowel syndrome’, *Alimentary Pharmacology and Therapeutics*, vol. 44, no. 7, pp. 693–703, 2016, doi: 10.1111/apt.13746.

[11] N. Nestel *et al.*, ‘The Gut Microbiome and Abiotic Factors as Potential Determinants of Postprandial Glucose Responses: A Single-Arm Meal Study’, *Frontiers in Nutrition*, vol. 7, 2021, Accessed: Feb. 17, 2023. [Online]. Available: https://www.frontiersin.org/articles/10.3389/fnut.2020.594850

[12] M. Müller, E. E. Canfora, and E. E. Blaak, ‘Gastrointestinal Transit Time, Glucose Homeostasis and Metabolic Health: Modulation by Dietary Fibers’, *Nutrients*, vol. 10, no. 3, p. 275, Feb. 2018, doi: 10.3390/nu10030275.

[13] M. A. Eastwood, J. A. Robertson, W. G. Brydon, and D. MacDonald, ‘Measurement of water-holding properties of fibre and their faecal bulking ability in man’, *British Journal of Nutrition*, vol. 50, no. 3, pp. 539–547, Nov. 1983, doi: 10.1079/BJN19830125.

[14] J. H. Cummings, M. Sc, and F. R. C. P. M. R. C. Dunn, ‘Constipation, dietary fibre and the control of large bowel function.’, *Postgraduate Medical Journal*, vol. 60, no. 709, p. 811, 1984, doi: 10.1136/PGMJ.60.709.811.

[15] G. V. Barbosa-Cánovas, A. J. Fontana, S. J. Schmidt, and T. P. Labuza, *Water activity in foods: Fundamentals and applications*. Blackwell Publishing Ltd, 2007, pp. 1–435. doi: 10.1002/9780470376454.

[16] D. Vandeputte, G. Falony, K. D’Hoe, S. Vieira-Silva, and J. Raes, ‘Water activity does not shape the microbiota in the human colon’, *Gut*, vol. 66, no. 10, pp. 1865–1866, Oct. 2017, doi: 10.1136/GUTJNL-2016-313530.

[17] T. Y. Wang, M. Liu, P. Portincasa, and D. Q. H. Wang, ‘New insights into the molecular mechanism of intestinal fatty acid absorption’, *European journal of clinical investigation*, vol. 43, no. 11, p. 1203, Nov. 2013, doi: 10.1111/ECI.12161.

[18] M. Müller *et al.*, ‘Distal colonic transit is linked to gut microbiota diversity and microbial fermentation in humans with slow colonic transit’, *American Journal of Physiology-Gastrointestinal and Liver Physiology*, vol. 318, no. 2, pp. G361–G369, Feb. 2020, doi: 10.1152/ajpgi.00283.2019.

[19] L. E. Oufir *et al.*, ‘Relations between transit time, fermentation products, and hydrogen consuming flora in healthy humans’, *Gut*, vol. 38, pp. 870–877, 1996, doi: 10.1136/gut.38.6.870.

[20] S. J. Lewis and K. W. Heaton, ‘Increasing butyrate concentration in the distal colon by accelerating intestinal transit’, *Gut*, vol. 41, no. 2, pp. 245–251, Aug. 1997, doi: 10.1136/GUT.41.2.245.

[21] J. H. Cummings, E. W. Pomare, W. J. Branch, C. P. Naylor, and G. T. Macfarlane, ‘Short chain fatty acids in human large intestine, portal, hepatic and venous blood.’, *Gut*, vol. 28, no. 10, pp. 1221–1227, Oct. 1987, doi: 10.1136/gut.28.10.1221.

[22] G. T. Macfarlane, G. R. Gibson, and J. H. Cummings, ‘Comparison of fermentation reactions in different regions of the human colon’, *The Journal of applied bacteriology*, vol. 72, no. 1, pp. 57–64, 1992, doi: 10.1111/J.1365-2672.1992.TB04882.X.

# Supplementary Figures


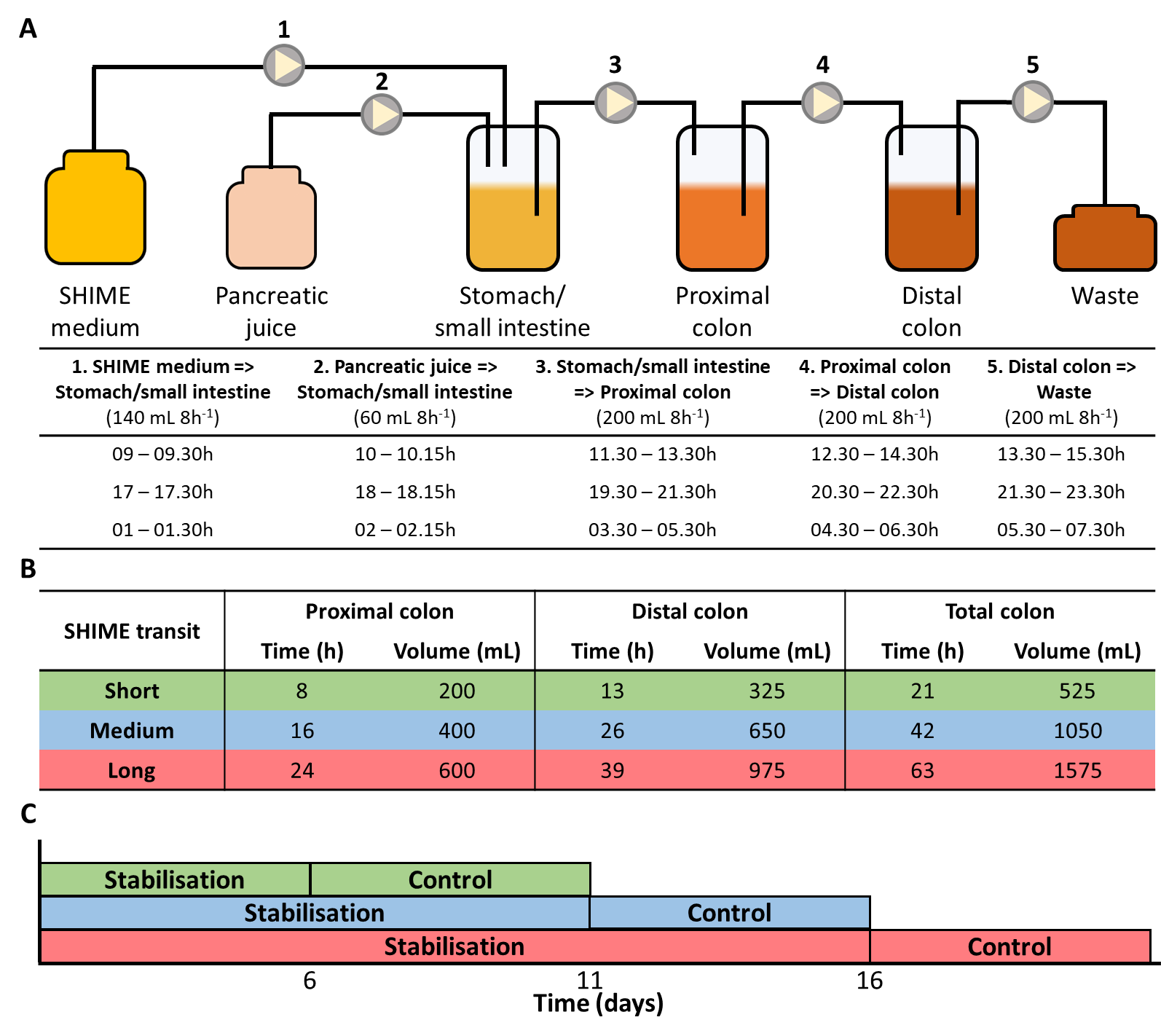


Figure S1: (**A)** The SHIME was semi-continuously fed 200mL of standardised nutritional SHIME medium (140mL, added in step 1) amended with pancreatic juice (60mL, added in step 2), of which the nutrient concentrations were adjusted to the proximal colon volumes to provide an equal nutrient concentration for every transit time configuration in eight-hour cycles (Table S3). (**B)** The residence time (h) in the SHIME was modified by adjusting the volumes of the colon compartments. Volumes were determined by multiplying the feed dosage rate (200mL 8h^-1^) with the corresponding transit time for every transit time configuration (short, medium, long). (**C)** The microbial ecosystem was considered stable after minimally 9 distal and 6 total colonic transit times, which corresponded to 6, 11 and 16 days for the short, medium and long transit time, respectively.


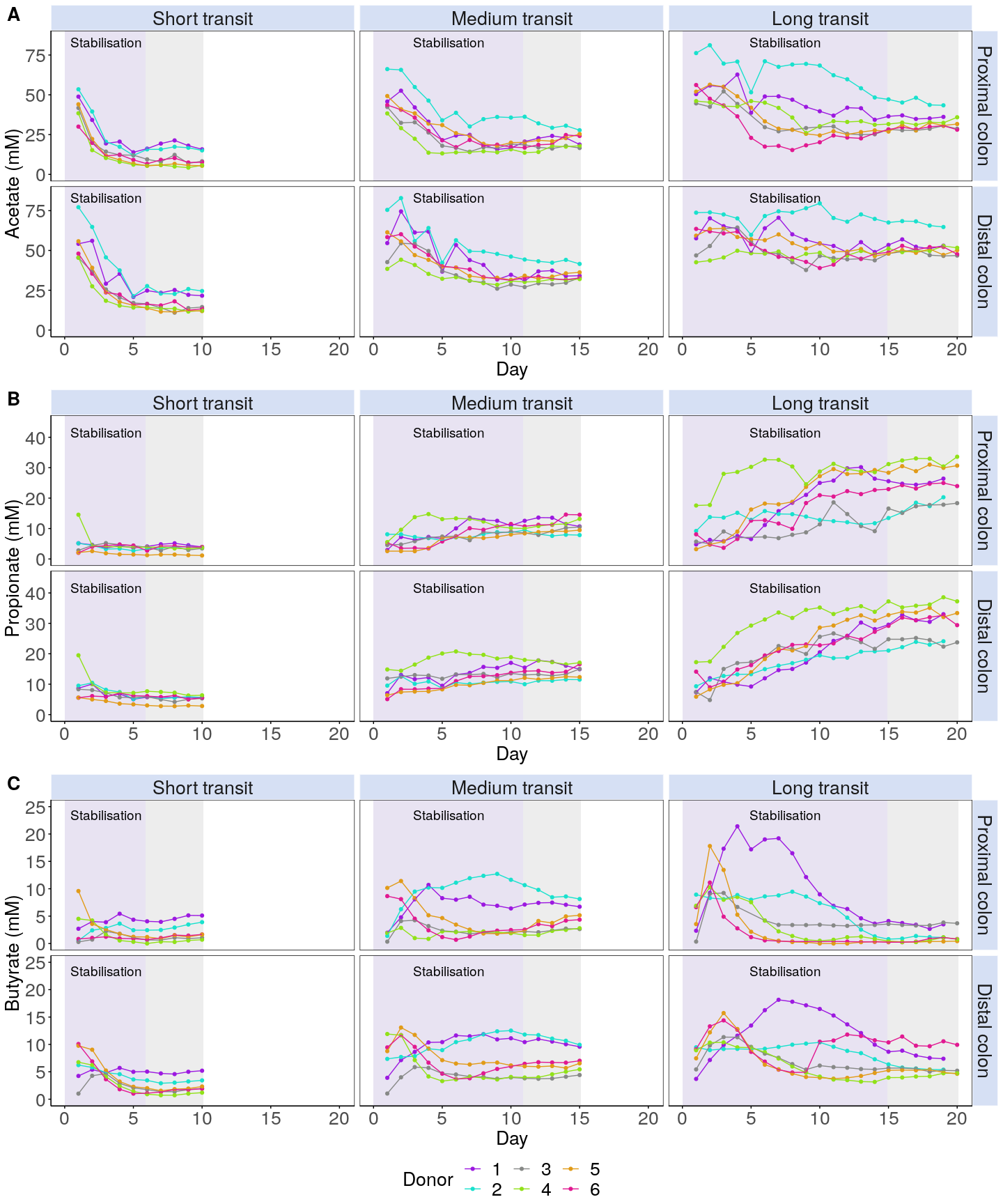


Figure S2: **(A)** Acetate, **(B)** propionate and **(C)** butyrate concentrations (mM) stabilised as a function of SHIME transit time for each faecal microbiome donor. The stabilisation period is highlighted in purple, thereafter the concentrations were considered stabilised, highlighted in grey. This corresponded to 6, 11 and 16 days for the short, medium and long transit time. Short, medium and long SHIME transit times were 8, 16 and 24h in the proximal colon and 13, 26 and 39h in the distal colon.


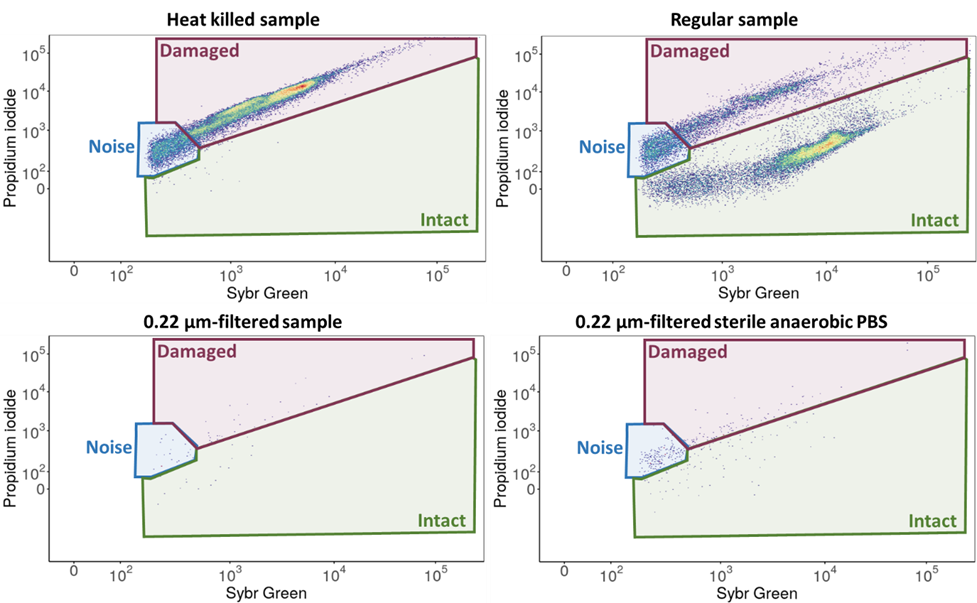


Figure S3: Gating strategy to delineate noise, damaged and intact cells. Heat killed samples were used to set the gate for the damaged cell population, regular samples were used to set the gate for the intact cell population and 0.22µm-filtered samples and anaerobic PBS diluent were used to set the noise gate.


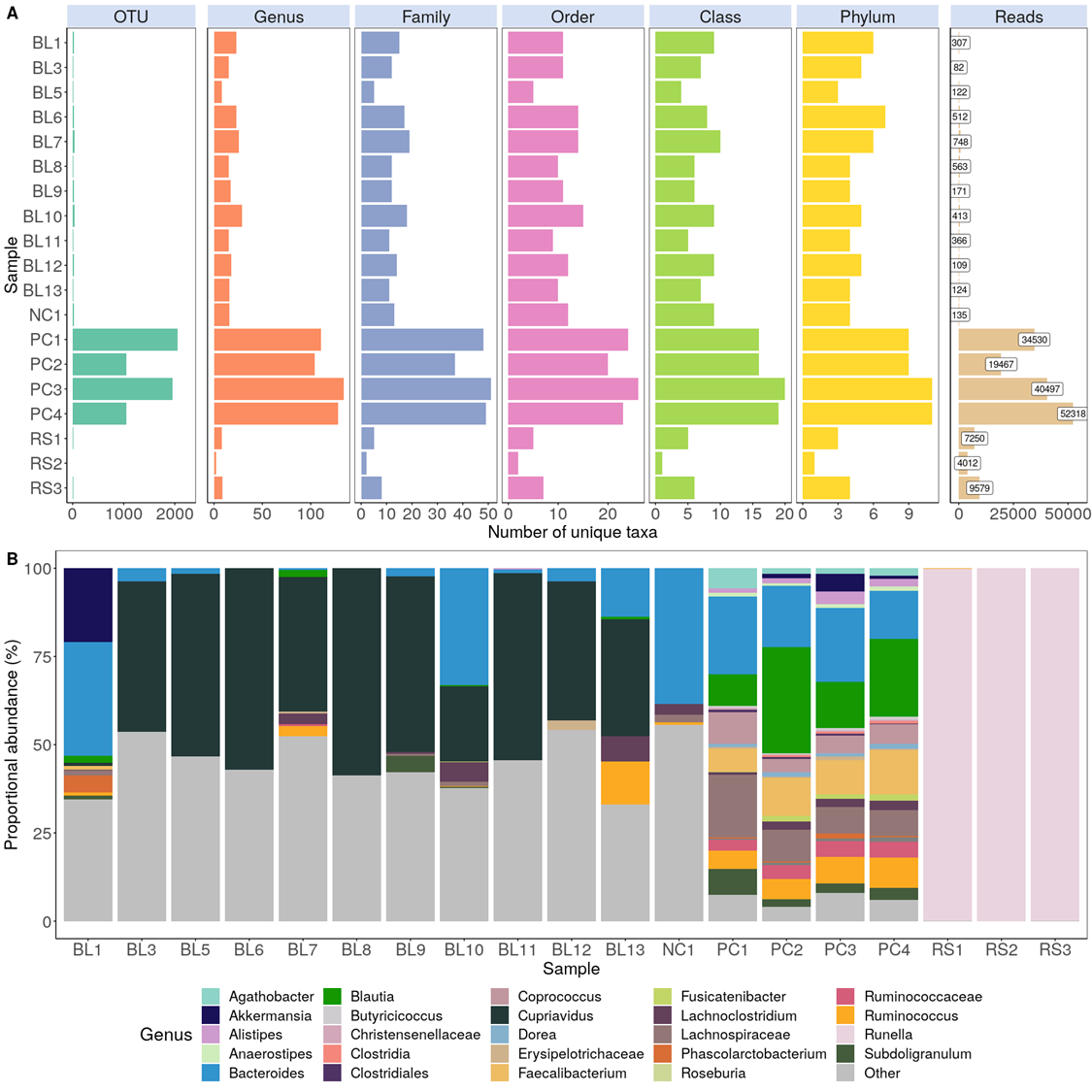


Figure S4: (**A**) Number of unique taxa in the 16S rRNA gene amplicon sequencing control samples. (**B**) Proportional abundance (%) of the 25 most abundant genera in the control samples. Less abundant genera are pooled into “Other”. Bl=DNA extraction blank, NC=Negative control with only the PCR reaction mixture, PC=positive control with a known composition, RS=pure *Runella slithyformis*. Higher level taxa are to be interpreted as unclassified genus belonging to the respective taxon.


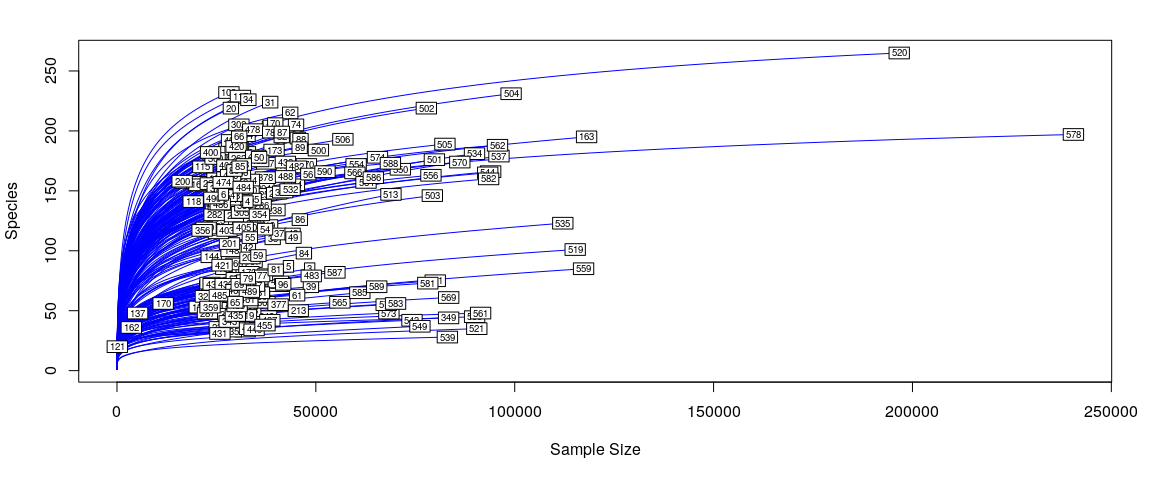


Figure S5: Rarefaction curves of 16S rRNA gene sequence count data. Sample number 121 (donor 2, distal colon, short SHIME transit , day 7) was removed from the dataset, due to insufficient reads (83), which falls in the range of the blanks and negative controls (Figure S4, max 748 read counts). The raw data corresponding to the sample identifiers can be found in the EBI ENA submission (ERP138715).


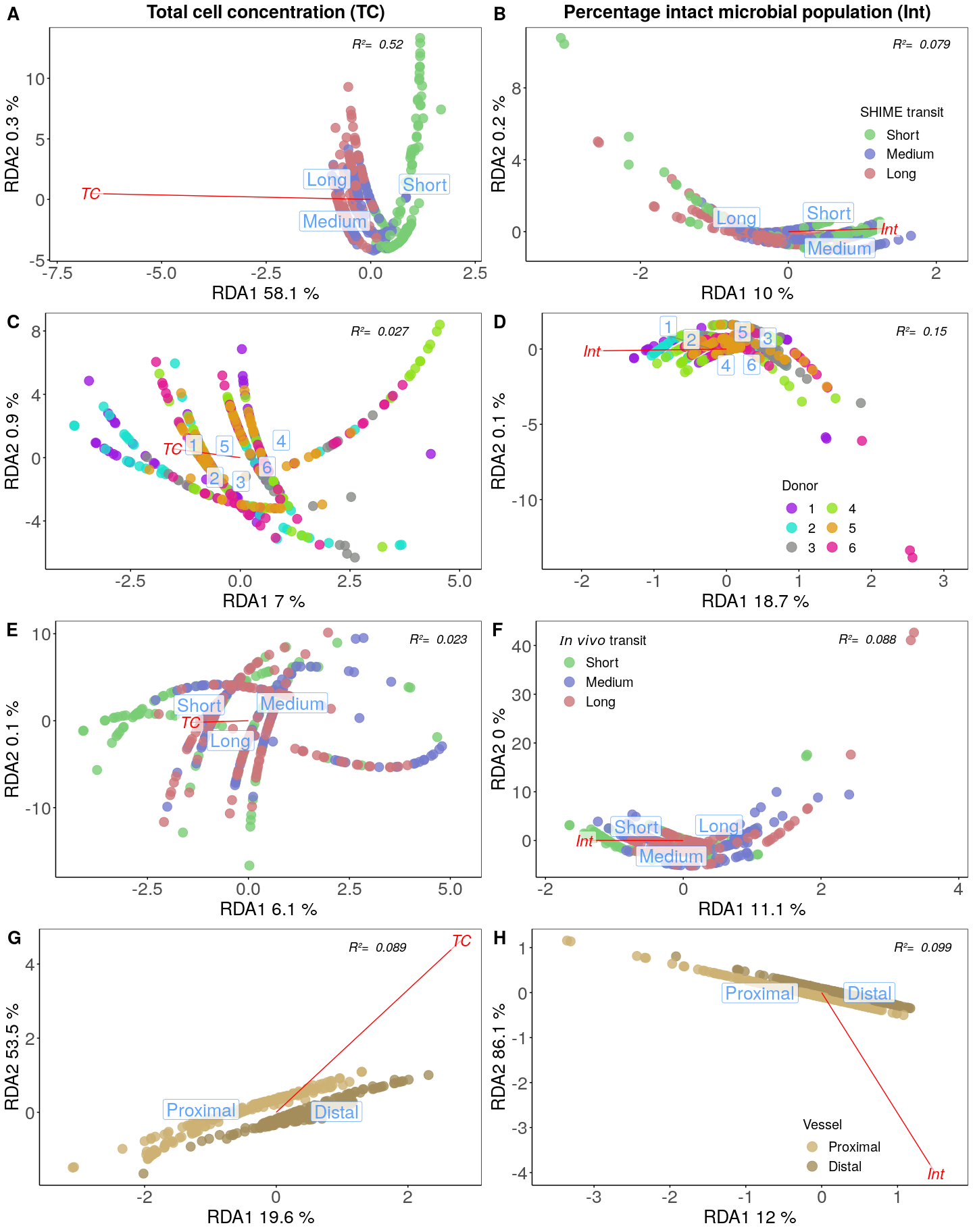


Figure S6: Distance based redundancy analysis correlation triplot with the response variable (TC=Total cell concentration, Int=Percentage of intact cells) shown in red and the centroid factor levels represented in blue (n=180). SHIME transit time (**A, B**), inter-individual variability (**C, D**), *in vivo* transit (**E, F**) and colon region (**G, H**) significantly (P_adjusted_=0.004) explain the variation in total microbial cell counts (A, C, E, G) and the percentage of intact microbial cells (B, D, F, H). The model fit is indicated by the adjusted R^2^ values depicted in the top right corner.


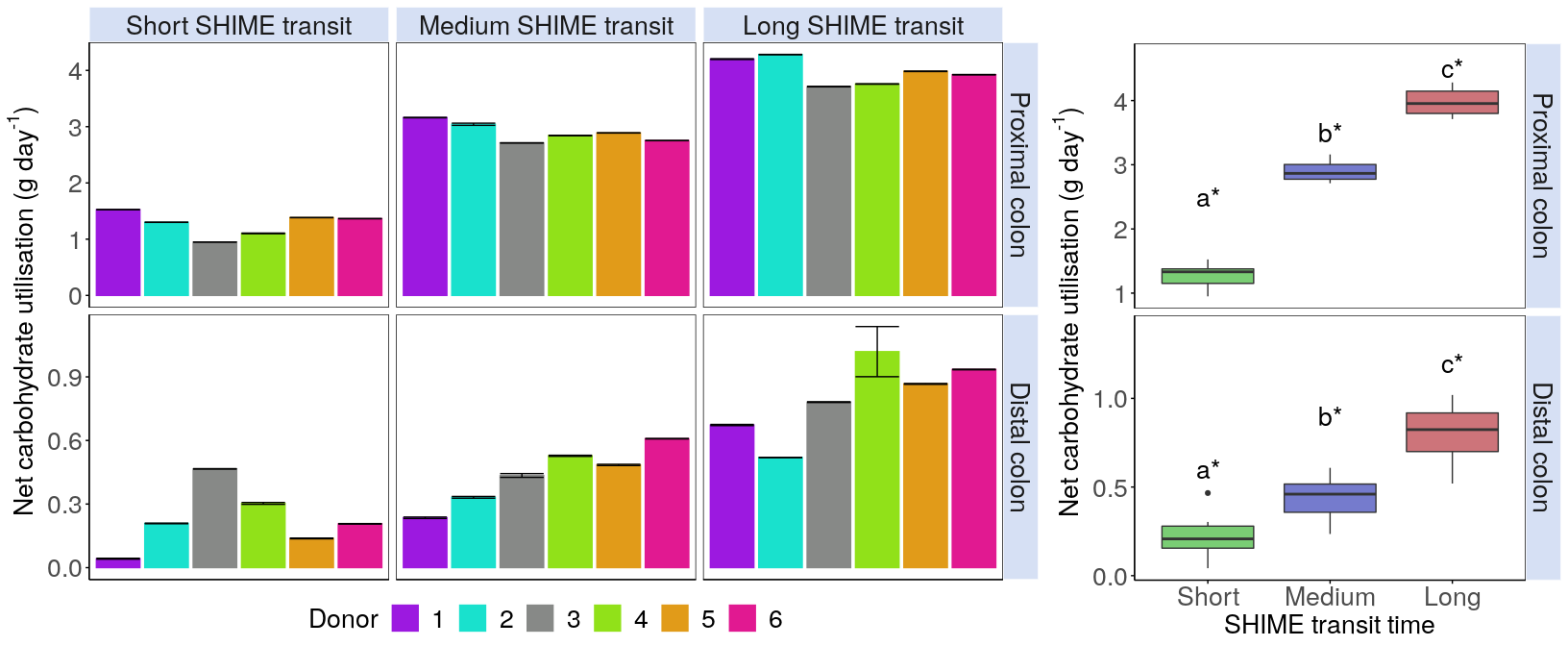


Figure S7: The net daily carbohydrate utilisation (g day^-1^) of the stabilised communities measured in triplicate in the SHIME’s proximal and distal colon vessels of six individual faecal microbiome donors shown separately (**A**) and grouped together (n=15) (**B**) increased as a function of SHIME transit time (n=90). Statistically significant differences between SHIME transit times are depicted by the letters a, b and c in panel B (unpaired two-sided Wilcoxon signed rank tests with Holm correction). Identical letters indicate no significant differences (P>0.05). Significant differences between colon regions of the same transit time are marked with asterisks (*) (P<0.05, paired two-sided Wilcoxon signed rank tests with Holm correction). Box plots display the interquartile range, median and outliers beyond the 1.5 times interquartile range (whiskers).


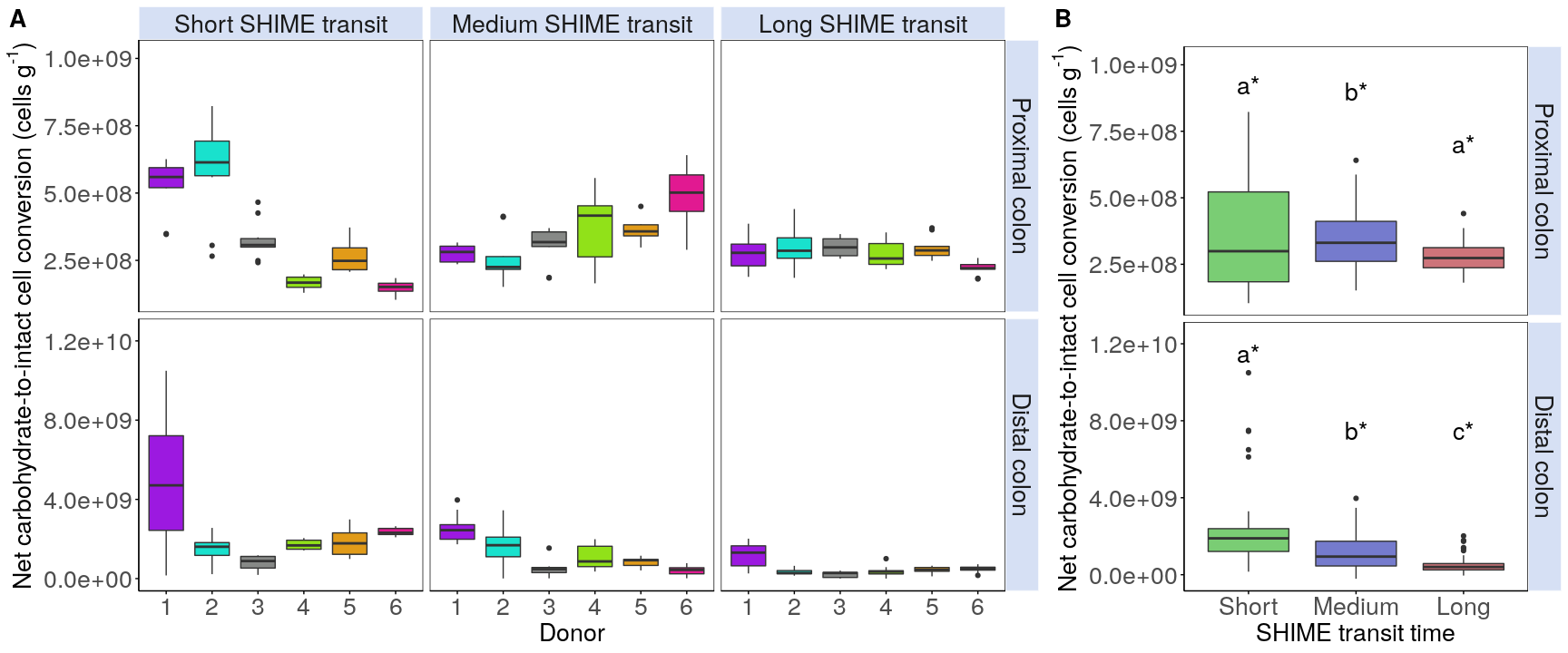


Figure S8: The net carbohydrate-to-intact cell conversion (cells g^-1^), i.e. the net intact cell production rate relative to the daily carbohydrate utilisation, of the stabilised communities in the SHIME’s proximal and distal colon vessels of six individual faecal microbiome donors shown separately (n=15) (**A**) and grouped together (n=90) (**B**) decreased as a function of SHIME transit time. Statistically significant differences between SHIME transit times are depicted by the letters a, b and c in panel B (unpaired two-sided Wilcoxon signed rank tests with Holm correction). Identical letters indicate no significant differences (P>0.05). Significant differences between colon regions of the same transit time are marked with asterisks (*) (P<0.05, paired two-sided Wilcoxon signed rank tests with Holm correction). Box plots display the interquartile range, median and outliers beyond the 1.5 times interquartile range (whiskers).


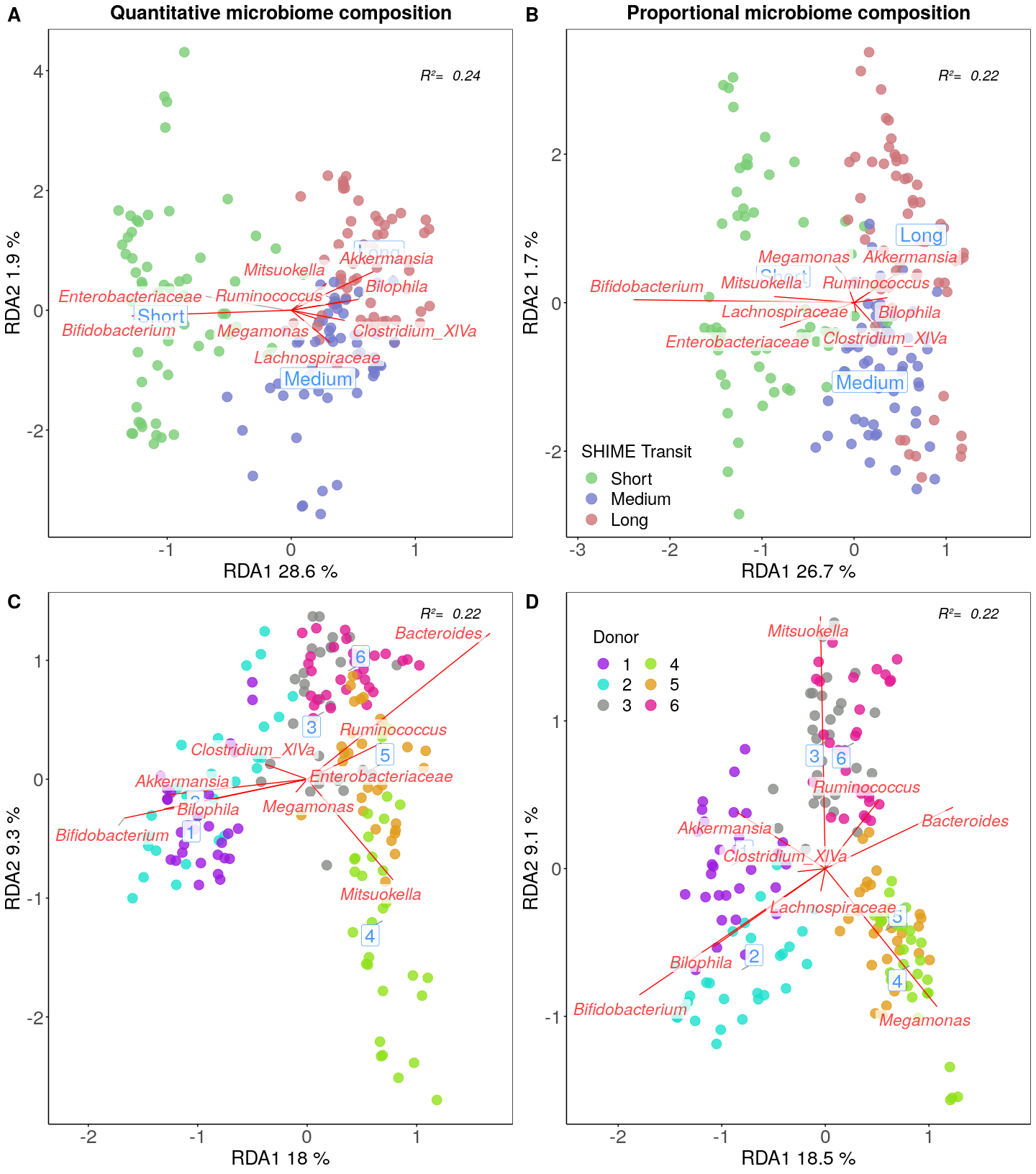


Figure S9: Distance based redundancy analysis correlation triplot with the 10 most abundant genera as response variables shown in red and the centroid factor levels represented in blue (n=90). SHIME transit time (**A, B**), inter-individual variability (**C, D**), *in vivo* transit (**E, F**) and colon region (**G, H**) significantly (P_adjusted_=0.004) explain the variation in quantitative genus level microbial community composition (A, C, E, G) and proportional genus level microbial community composition (B, D, F, H). The model fit is indicated by the adjusted R^2^ values depicted in the top right corner. Higher level taxa are to be interpreted as unclassified genus belonging to the respective taxon.


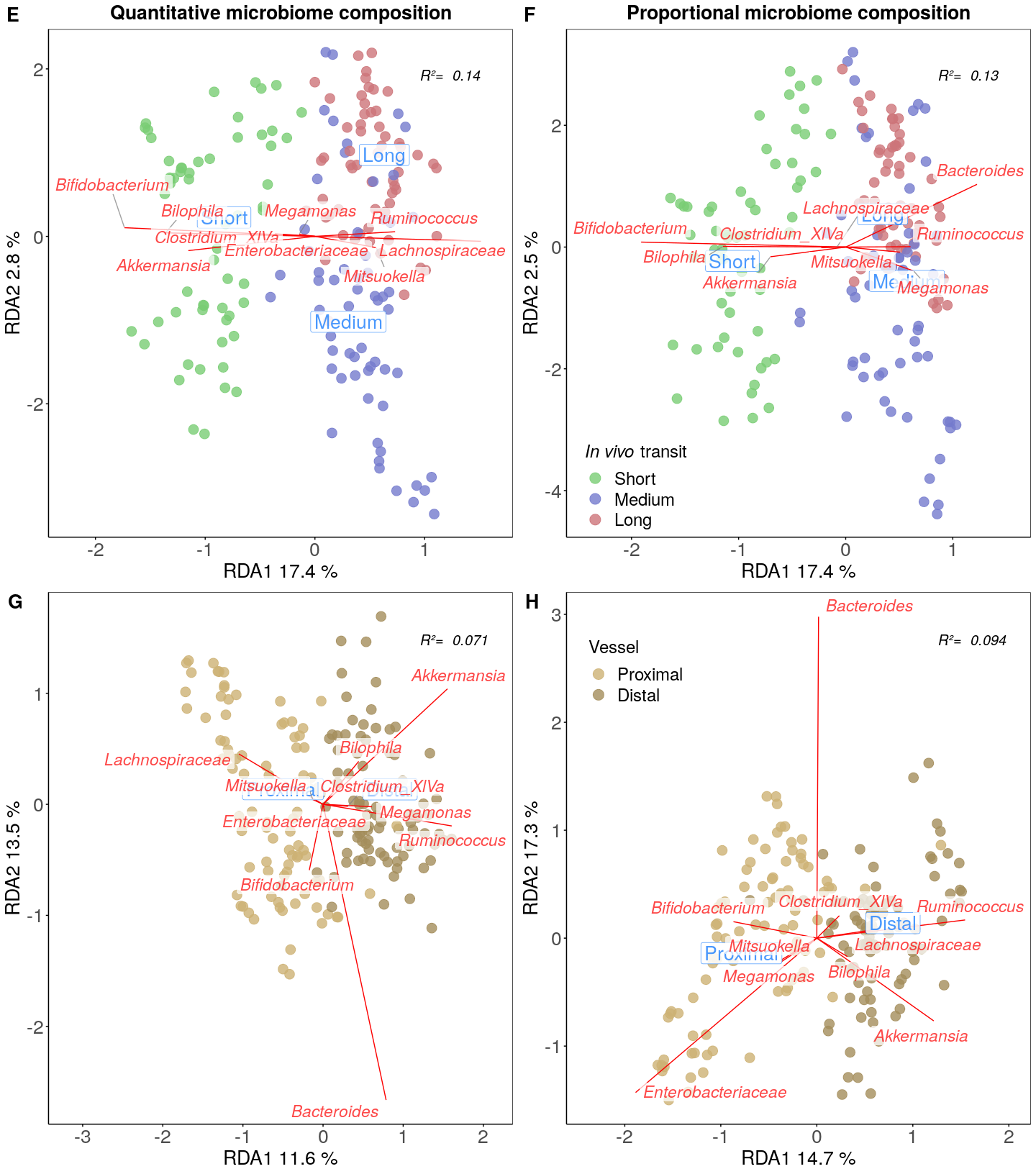


Figure S9 – Continued: Distance based redundancy analysis correlation triplot with the 10 most abundant genera as response variables shown in red and the centroid factor levels represented in blue (n=90). SHIME transit time (**A, B**), inter-individual variability (**C, D**), *in vivo* transit (**E, F**) and colon region (**G, H**) significantly (P_adjusted_=0.004) explain the variation in quantitative genus level microbial community composition (A, C, E, G) and proportional genus level microbial community composition (B, D, F, H). The model fit is indicated by the adjusted R^2^ values depicted in the top right corner. Higher level taxa are to be interpreted as unclassified genus belonging to the respective taxon.


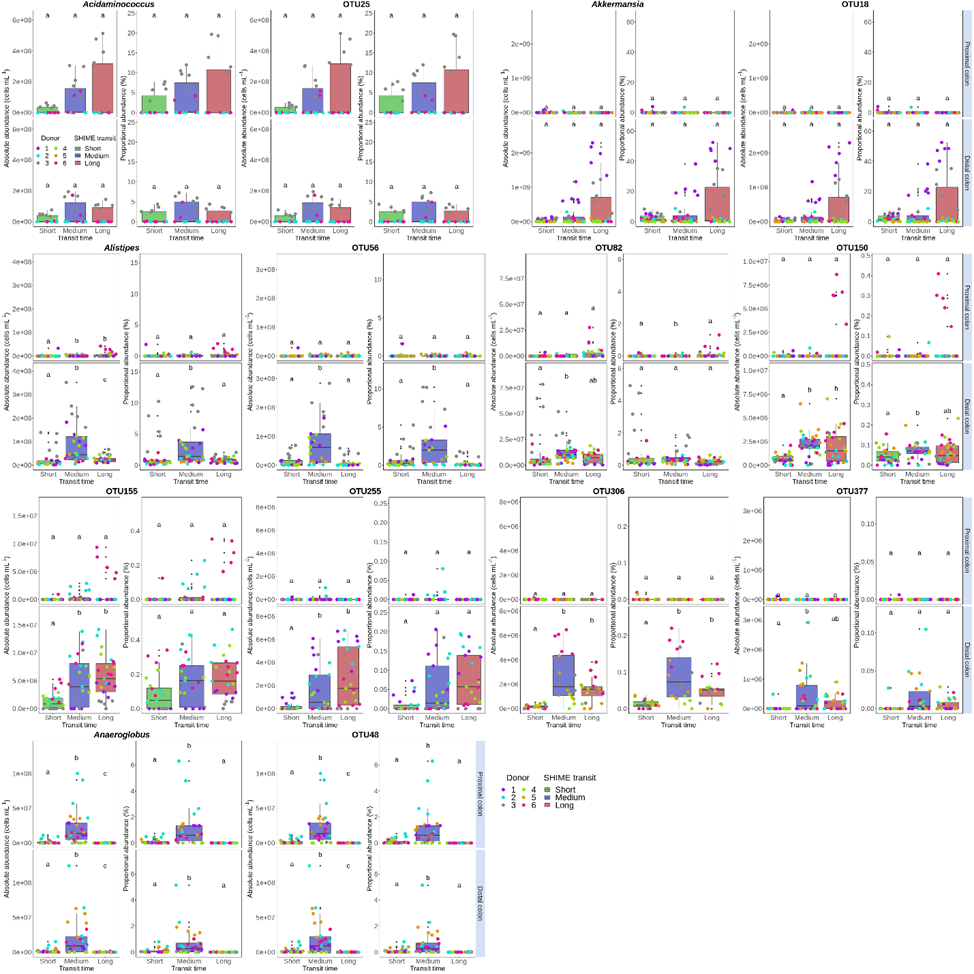


Figure S10: Absolute (cells mL^-1^) and proportional abundances (%) of the top 31 most abundant genera and corresponding OTUs as a function of SHIME transit time in the proximal and distal colon region (n=30). Only significant OTUs with a relative abundance of more than 5% within the genus were shown. Short, medium and long SHIME transit times were 8, 16 and 24h in the proximal colon and 13, 26 and 39h in the distal colon. Statistically significant differences between SHIME transit times are indicated by the letters a, b and c (unpaired two-sided Wilcoxon signed rank tests with Holm correction). Identical letters indicate no significant differences (P>0.05). Significant differences between colon regions are indicated with asterisks (*) (P<0.05, paired two-sided Wilcoxon signed rank tests with Holm correction). Higher level taxa are to be interpreted as unclassified genus belonging to the respective taxon. Box plots display individual data points, as well as the interquartile range, median and outliers beyond the 1.5 times interquartile range (whiskers).


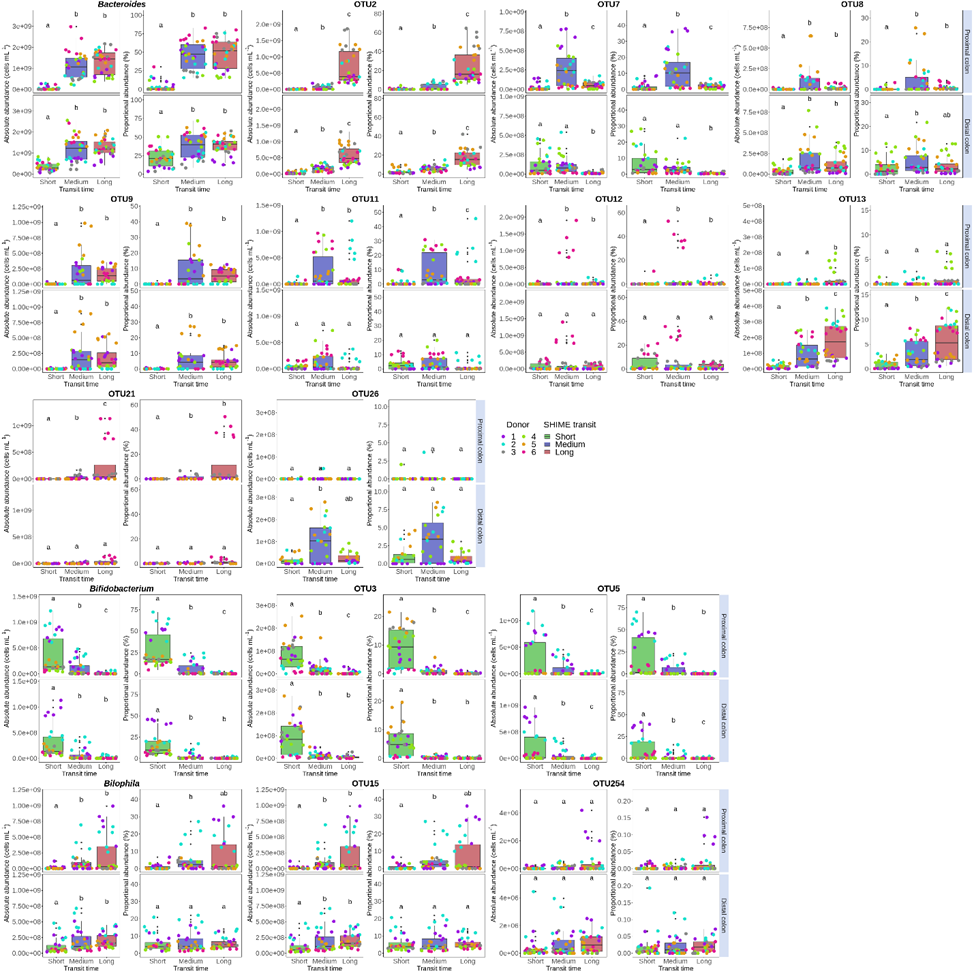


Figure S10 – Continued: Absolute (cells mL^-1^) and proportional abundances (%) of the top 31 most abundant genera and corresponding OTUs as a function of SHIME transit time in the proximal and distal colon region (n=30). Only significant OTUs with a relative abundance of more than 5% within the genus were shown. Short, medium and long SHIME transit times were 8, 16 and 24h in the proximal colon and 13, 26 and 39h in the distal colon. Statistically significant differences between SHIME transit times are indicated by the letters a, b and c (unpaired two-sided Wilcoxon signed rank tests with Holm correction). Identical letters indicate no significant differences (P>0.05). Significant differences between colon regions are indicated with asterisks (*) (P<0.05, paired two-sided Wilcoxon signed rank tests with Holm correction). Higher level taxa are to be interpreted as unclassified genus belonging to the respective taxon. Box plots display individual data points, as well as the interquartile range, median and outliers beyond the 1.5 times interquartile range (whiskers).


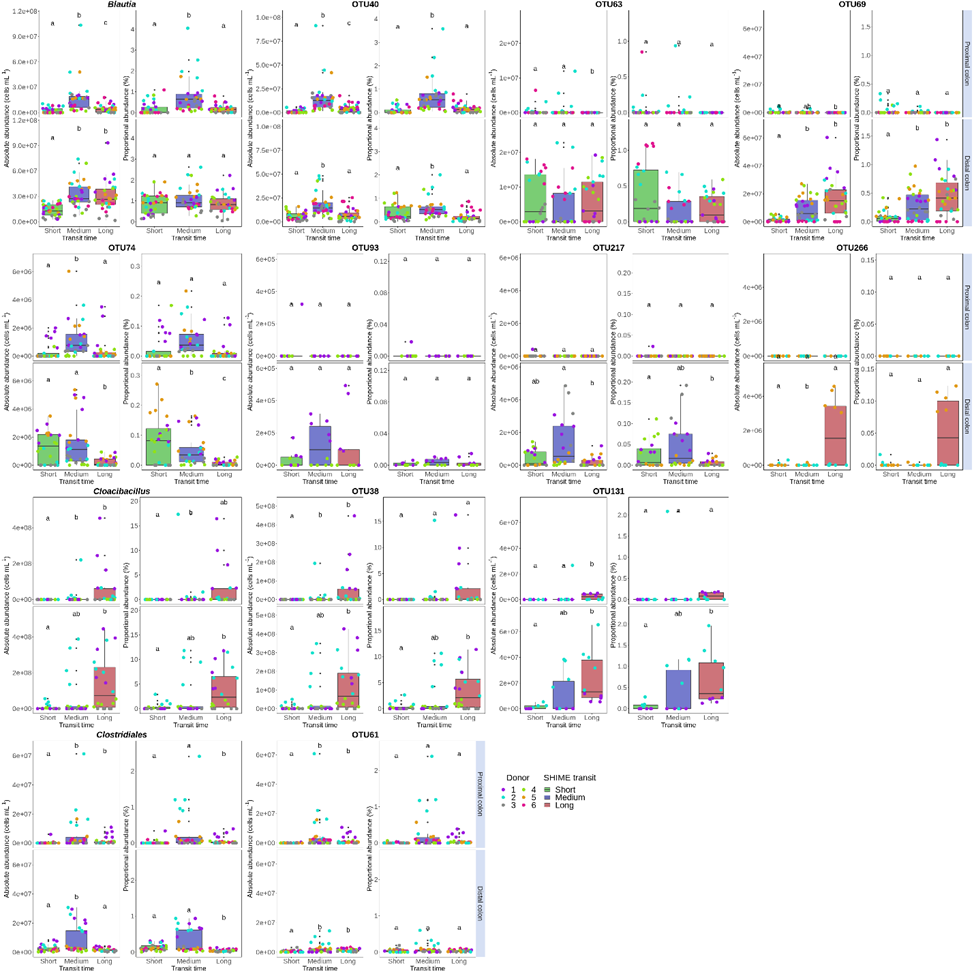


Figure S10 – Continued: Absolute (cells mL^-1^) and proportional abundances (%) of the top 31 most abundant genera and corresponding OTUs as a function of SHIME transit time in the proximal and distal colon region (n=30). Only significant OTUs with a relative abundance of more than 5% within the genus were shown. Short, medium and long SHIME transit times were 8, 16 and 24h in the proximal colon and 13, 26 and 39h in the distal colon. Statistically significant differences between SHIME transit times are indicated by the letters a, b and c (unpaired two-sided Wilcoxon signed rank tests with Holm correction). Identical letters indicate no significant differences (P>0.05). Significant differences between colon regions are indicated with asterisks (*) (P<0.05, paired two-sided Wilcoxon signed rank tests with Holm correction). Higher level taxa are to be interpreted as unclassified genus belonging to the respective taxon. Box plots display individual data points, as well as the interquartile range, median and outliers beyond the 1.5 times interquartile range (whiskers).


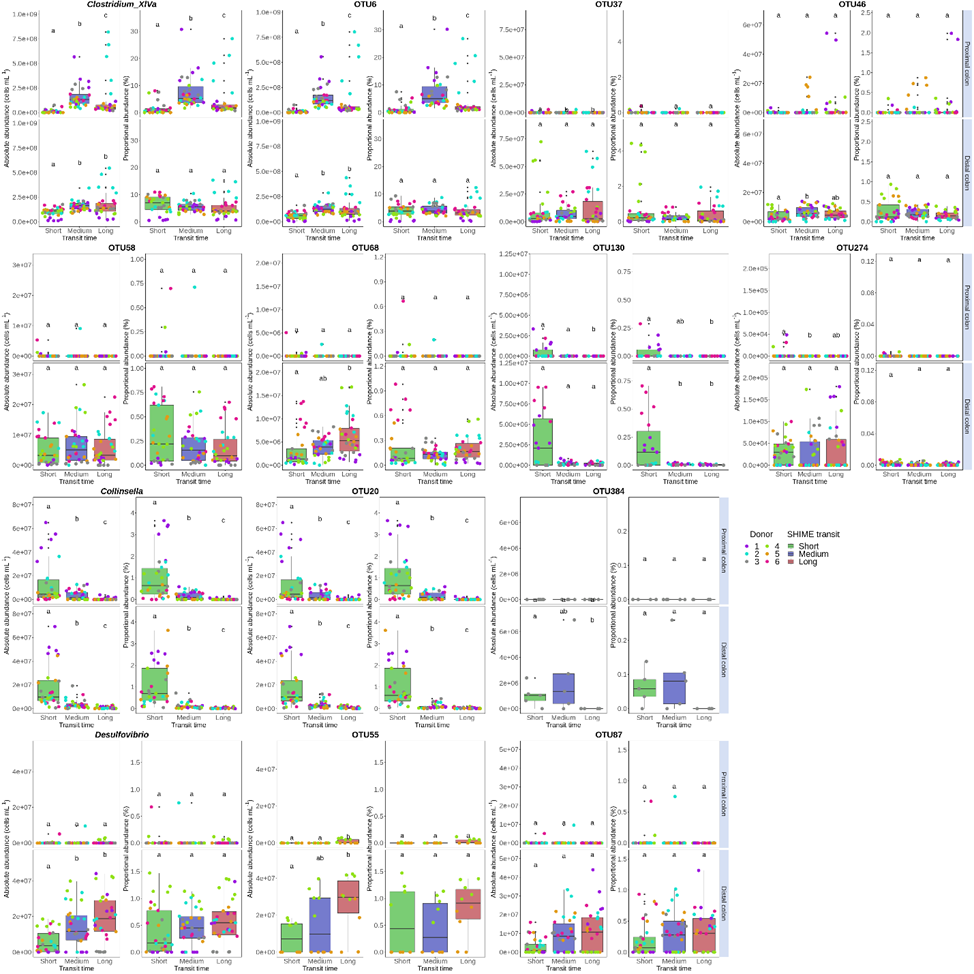


Figure S10 – Continued: Absolute (cells mL^-1^) and proportional abundances (%) of the top 31 most abundant genera and corresponding OTUs as a function of SHIME transit time in the proximal and distal colon region (n=30). Only significant OTUs with a relative abundance of more than 5% within the genus were shown. Short, medium and long SHIME transit times were 8, 16 and 24h in the proximal colon and 13, 26 and 39h in the distal colon. Statistically significant differences between SHIME transit times are indicated by the letters a, b and c (unpaired two-sided Wilcoxon signed rank tests with Holm correction). Identical letters indicate no significant differences (P>0.05). Significant differences between colon regions are indicated with asterisks (*) (P<0.05, paired two-sided Wilcoxon signed rank tests with Holm correction). Higher level taxa are to be interpreted as unclassified genus belonging to the respective taxon. Box plots display individual data points, as well as the interquartile range, median and outliers beyond the 1.5 times interquartile range (whiskers).


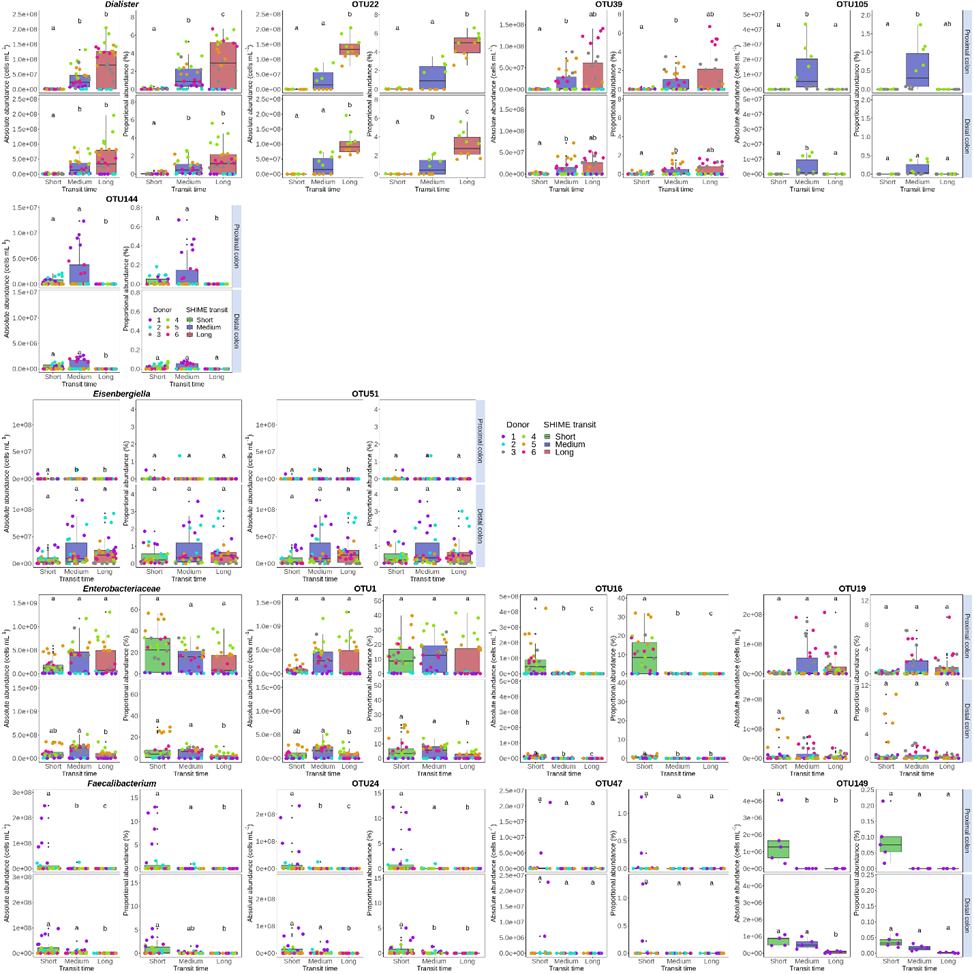


Figure S10 – Continued: Absolute (cells mL^-1^) and proportional abundances (%) of the top 31 most abundant genera and corresponding OTUs as a function of SHIME transit time in the proximal and distal colon region (n=30). Only significant OTUs with a relative abundance of more than 5% within the genus were shown. Short, medium and long SHIME transit times were 8, 16 and 24h in the proximal colon and 13, 26 and 39h in the distal colon. Statistically significant differences between SHIME transit times are indicated by the letters a, b and c (unpaired two-sided Wilcoxon signed rank tests with Holm correction). Identical letters indicate no significant differences (P>0.05). Significant differences between colon regions are indicated with asterisks (*) (P<0.05, paired two-sided Wilcoxon signed rank tests with Holm correction). Higher level taxa are to be interpreted as unclassified genus belonging to the respective taxon. Box plots display individual data points, as well as the interquartile range, median and outliers beyond the 1.5 times interquartile range (whiskers).


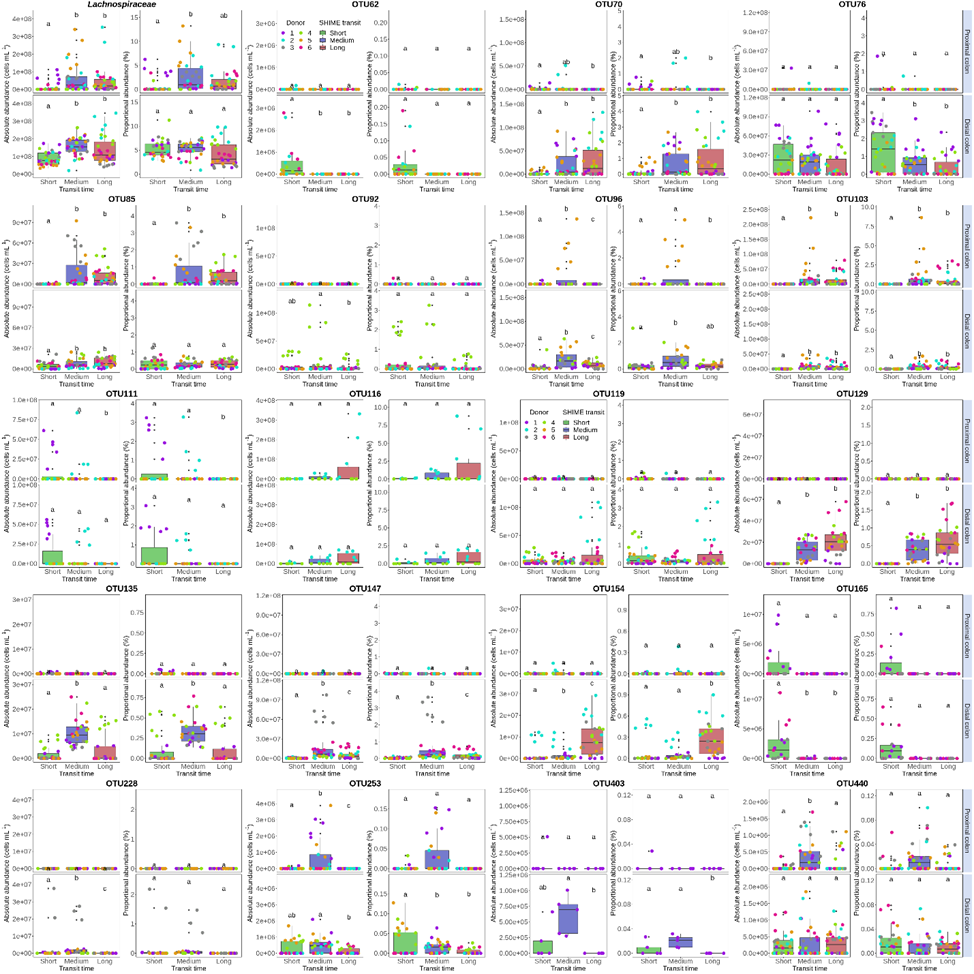


Figure S10 – Continued: Absolute (cells mL^-1^) and proportional abundances (%) of the top 31 most abundant genera and corresponding OTUs as a function of SHIME transit time in the proximal and distal colon region (n=30). Only significant OTUs with a relative abundance of more than 5% within the genus were shown. Short, medium and long SHIME transit times were 8, 16 and 24h in the proximal colon and 13, 26 and 39h in the distal colon. Statistically significant differences between SHIME transit times are indicated by the letters a, b and c (unpaired two-sided Wilcoxon signed rank tests with Holm correction). Identical letters indicate no significant differences (P>0.05). Significant differences between colon regions are indicated with asterisks (*) (P<0.05, paired two-sided Wilcoxon signed rank tests with Holm correction). Higher level taxa are to be interpreted as unclassified genus belonging to the respective taxon. Box plots display individual data points, as well as the interquartile range, median and outliers beyond the 1.5 times interquartile range (whiskers).


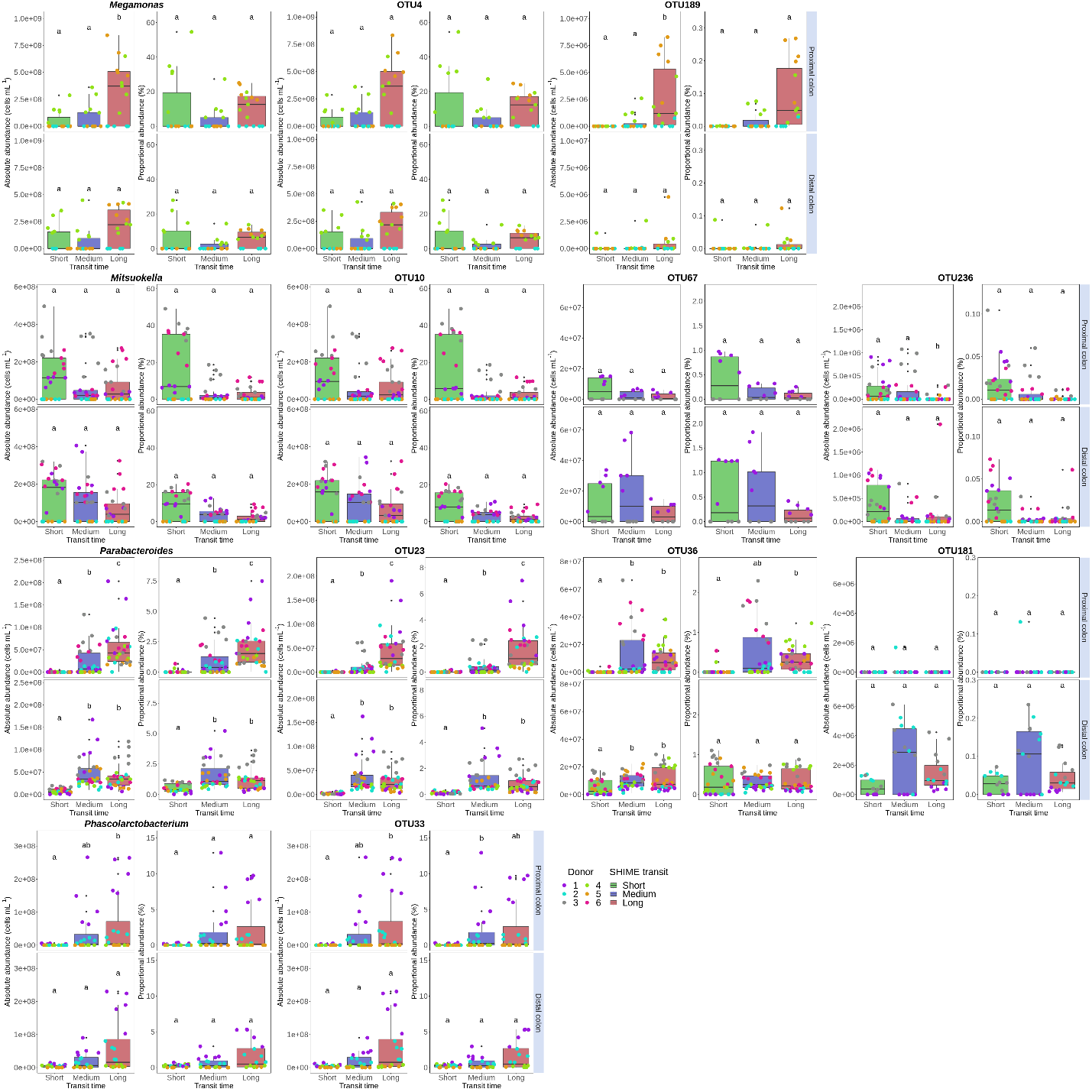


Figure S10 – Continued: Absolute (cells mL^-1^) and proportional abundances (%) of the top 31 most abundant genera and corresponding OTUs as a function of SHIME transit time in the proximal and distal colon region (n=30). Only significant OTUs with a relative abundance of more than 5% within the genus were shown. Short, medium and long SHIME transit times were 8, 16 and 24h in the proximal colon and 13, 26 and 39h in the distal colon. Statistically significant differences between SHIME transit times are indicated by the letters a, b and c (unpaired two-sided Wilcoxon signed rank tests with Holm correction). Identical letters indicate no significant differences (P>0.05). Significant differences between colon regions are indicated with asterisks (*) (P<0.05, paired two-sided Wilcoxon signed rank tests with Holm correction). Higher level taxa are to be interpreted as unclassified genus belonging to the respective taxon. Box plots display individual data points, as well as the interquartile range, median and outliers beyond the 1.5 times interquartile range (whiskers).


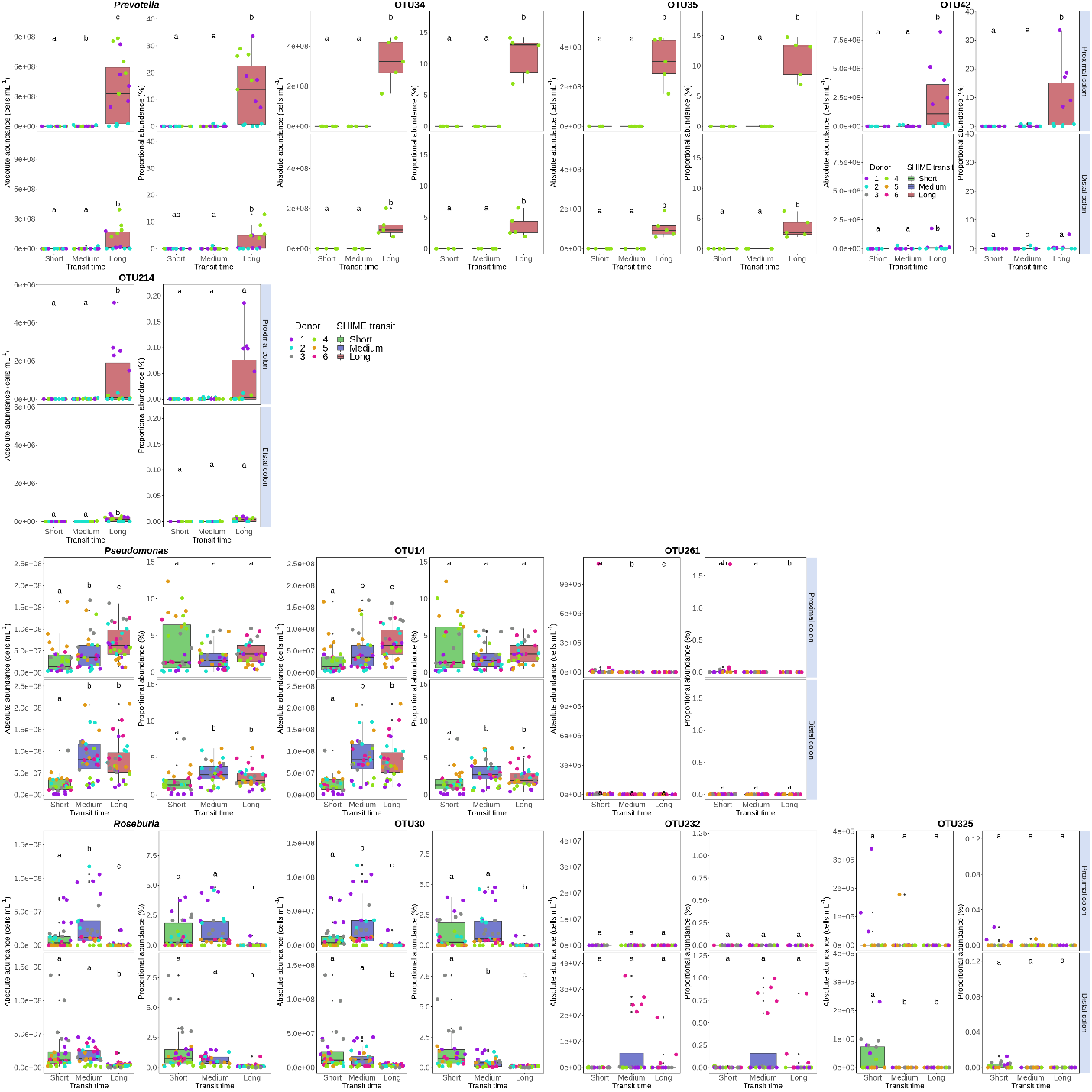


Figure S10 – Continued: Absolute (cells mL^-1^) and proportional abundances (%) of the top 31 most abundant genera and corresponding OTUs as a function of SHIME transit time in the proximal and distal colon region (n=30). Only significant OTUs with a relative abundance of more than 5% within the genus were shown. Short, medium and long SHIME transit times were 8, 16 and 24h in the proximal colon and 13, 26 and 39h in the distal colon. Statistically significant differences between SHIME transit times are indicated by the letters a, b and c (unpaired two-sided Wilcoxon signed rank tests with Holm correction). Identical letters indicate no significant differences (P>0.05). Significant differences between colon regions are indicated with asterisks (*) (P<0.05, paired two-sided Wilcoxon signed rank tests with Holm correction). Higher level taxa are to be interpreted as unclassified genus belonging to the respective taxon. Box plots display individual data points, as well as the interquartile range, median and outliers beyond the 1.5 times interquartile range (whiskers).


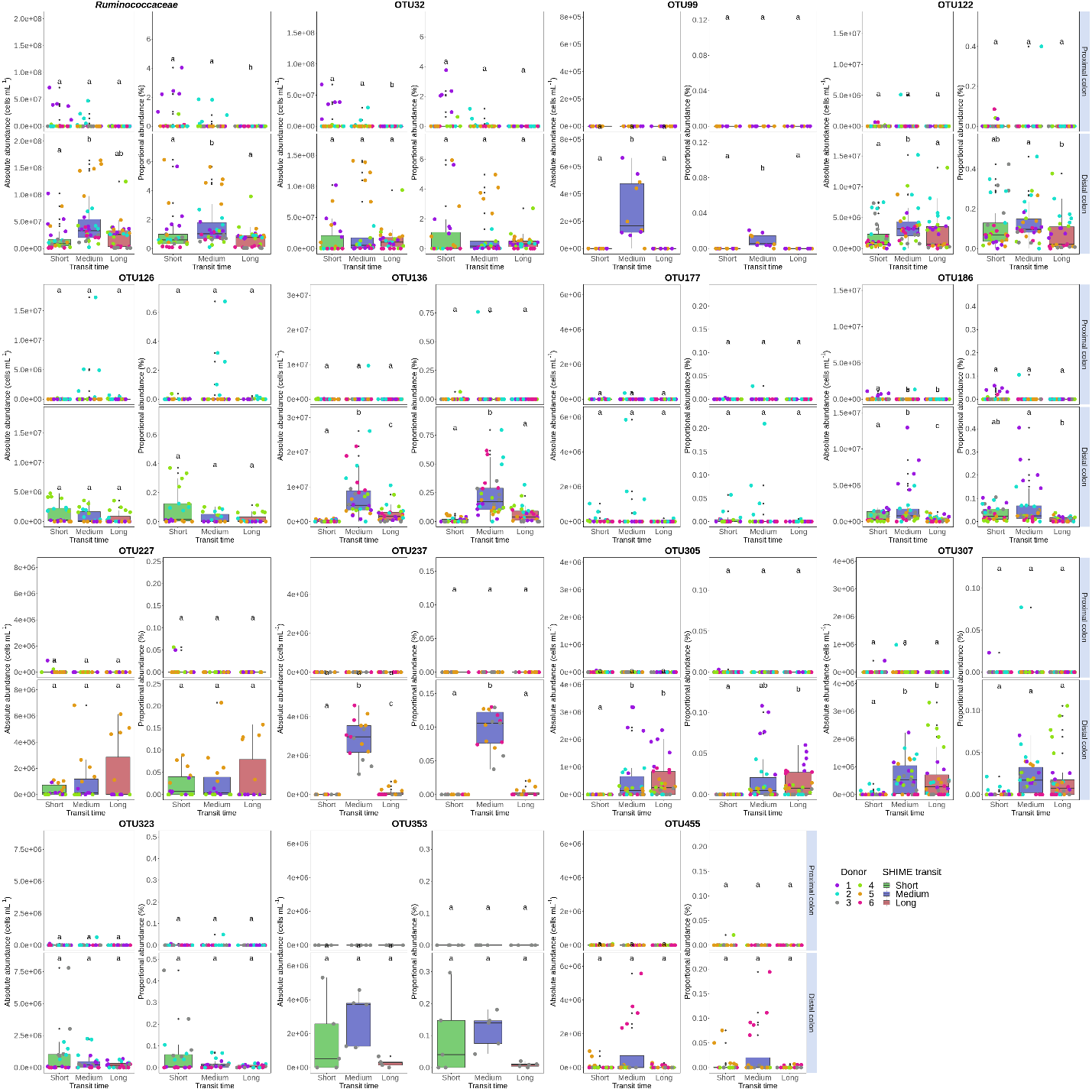


Figure S10 – Continued: Absolute (cells mL^-1^) and proportional abundances (%) of the top 31 most abundant genera and corresponding OTUs as a function of SHIME transit time in the proximal and distal colon region (n=30). Only significant OTUs with a relative abundance of more than 5% within the genus were shown. Short, medium and long SHIME transit times were 8, 16 and 24h in the proximal colon and 13, 26 and 39h in the distal colon. Statistically significant differences between SHIME transit times are indicated by the letters a, b and c (unpaired two-sided Wilcoxon signed rank tests with Holm correction). Identical letters indicate no significant differences (P>0.05). Significant differences between colon regions are indicated with asterisks (*) (P<0.05, paired two-sided Wilcoxon signed rank tests with Holm correction). Higher level taxa are to be interpreted as unclassified genus belonging to the respective taxon. Box plots display individual data points, as well as the interquartile range, median and outliers beyond the 1.5 times interquartile range (whiskers).


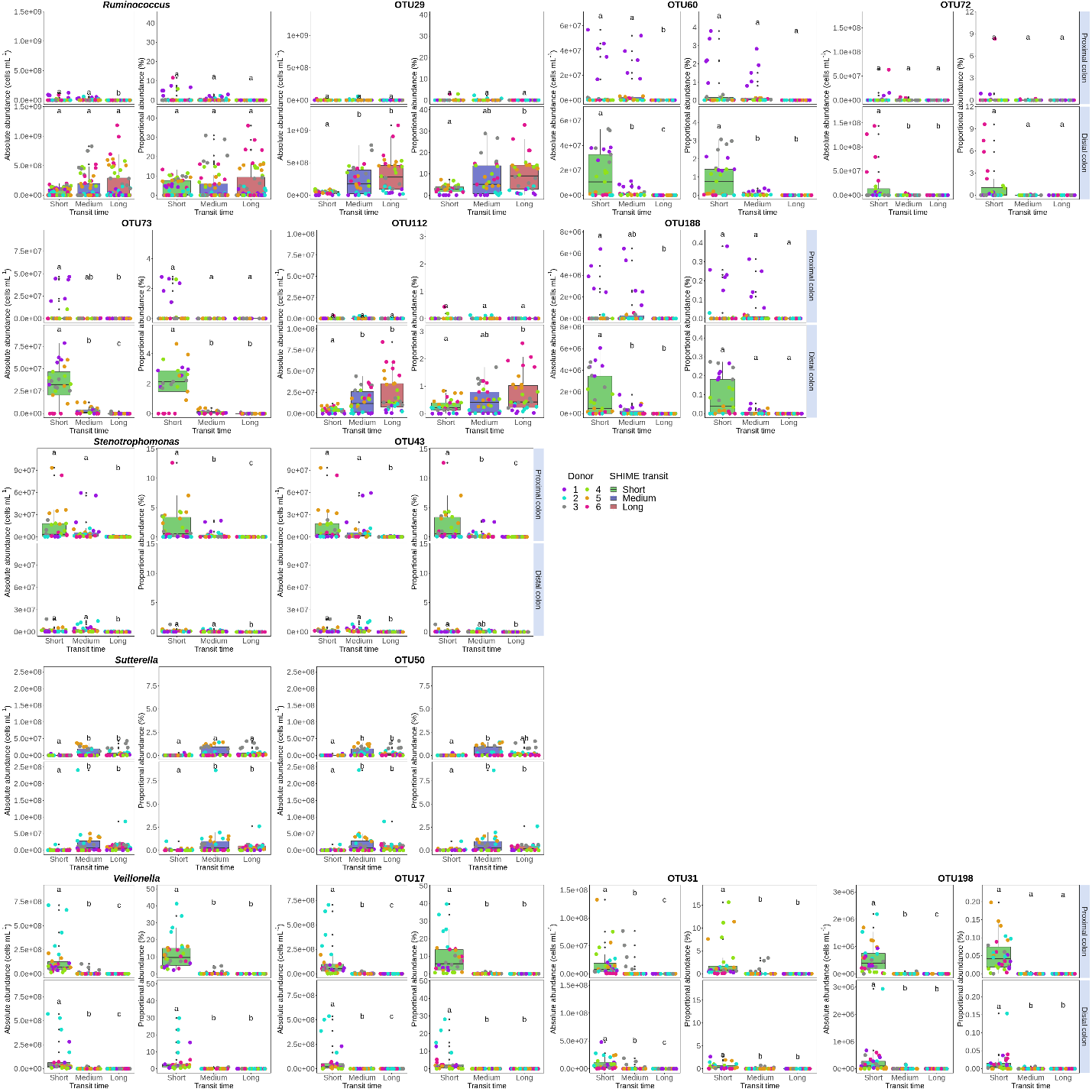


Figure S10 – Continued: Absolute (cells mL^-1^) and proportional abundances (%) of the top 31 most abundant genera and corresponding OTUs as a function of SHIME transit time in the proximal and distal colon region (n=30). Only significant OTUs with a relative abundance of more than 5% within the genus were shown. Short, medium and long SHIME transit times were 8, 16 and 24h in the proximal colon and 13, 26 and 39h in the distal colon. Statistically significant differences between SHIME transit times are indicated by the letters a, b and c (unpaired two-sided Wilcoxon signed rank tests with Holm correction). Identical letters indicate no significant differences (P>0.05). Significant differences between colon regions are indicated with asterisks (*) (P<0.05, paired two-sided Wilcoxon signed rank tests with Holm correction). Higher level taxa are to be interpreted as unclassified genus belonging to the respective taxon. Box plots display individual data points, as well as the interquartile range, median and outliers beyond the 1.5 times interquartile range (whiskers).


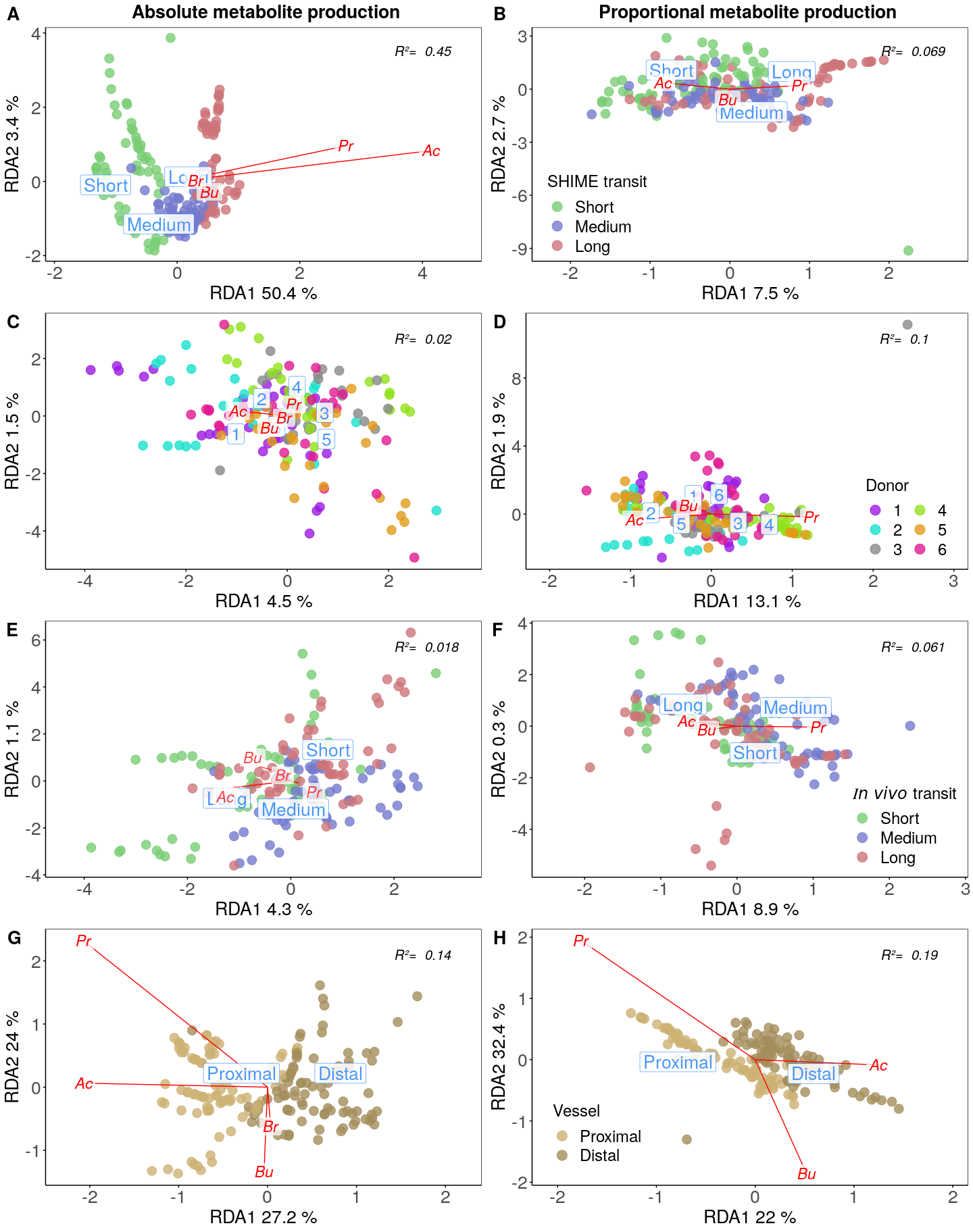


Figure S11: Redundancy analysis correlation triplot with metabolites as response variables (Ac=acetate, Pr=propionate, Bu=butyrate, Br=branched SCFA) shown in red and the centroid factor levels represented in blue (n=90). SHIME transit time (**A, B**), inter-individual variability (**C, D**), *in vivo* transit (**E, F**) and colon region (**G, H**) significantly (P_adjusted_=0.004) explain the variation in absolute metabolic net production (A, C, E, G) and proportional net production (B, D, F, H). The model fit is indicated by the adjusted R^2^ values, depicted in the top right corner.


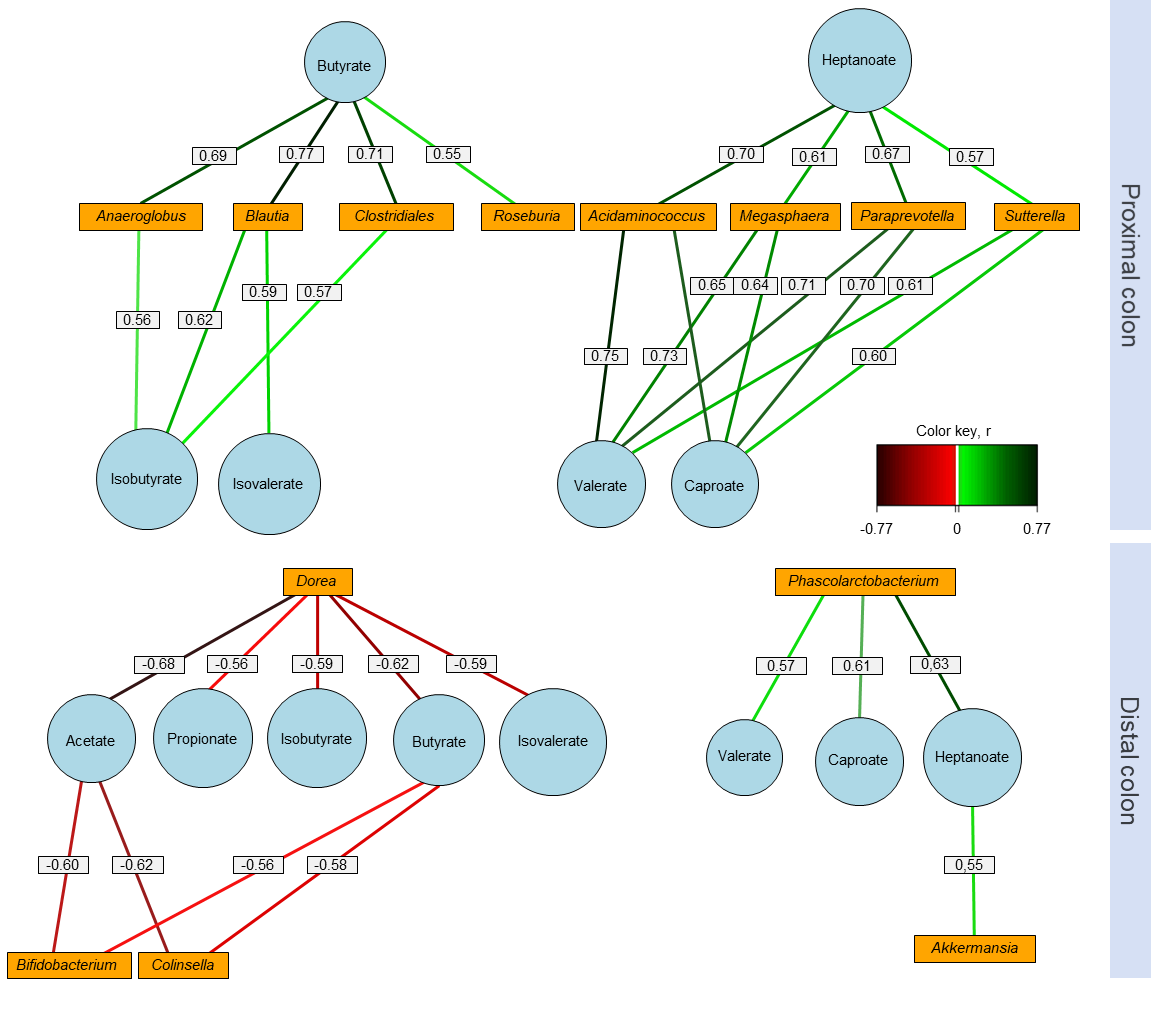


Figure S12: Relevance Networks based on sPLS regressions reveal correlations (>0.55) between specific genera and net SCFA production. Net SCFA production nodes are depicted as light blue circles and absolute genera abundances are depicted as orange rectangles. Negative correlations are depicted in red and positive correlations in green.


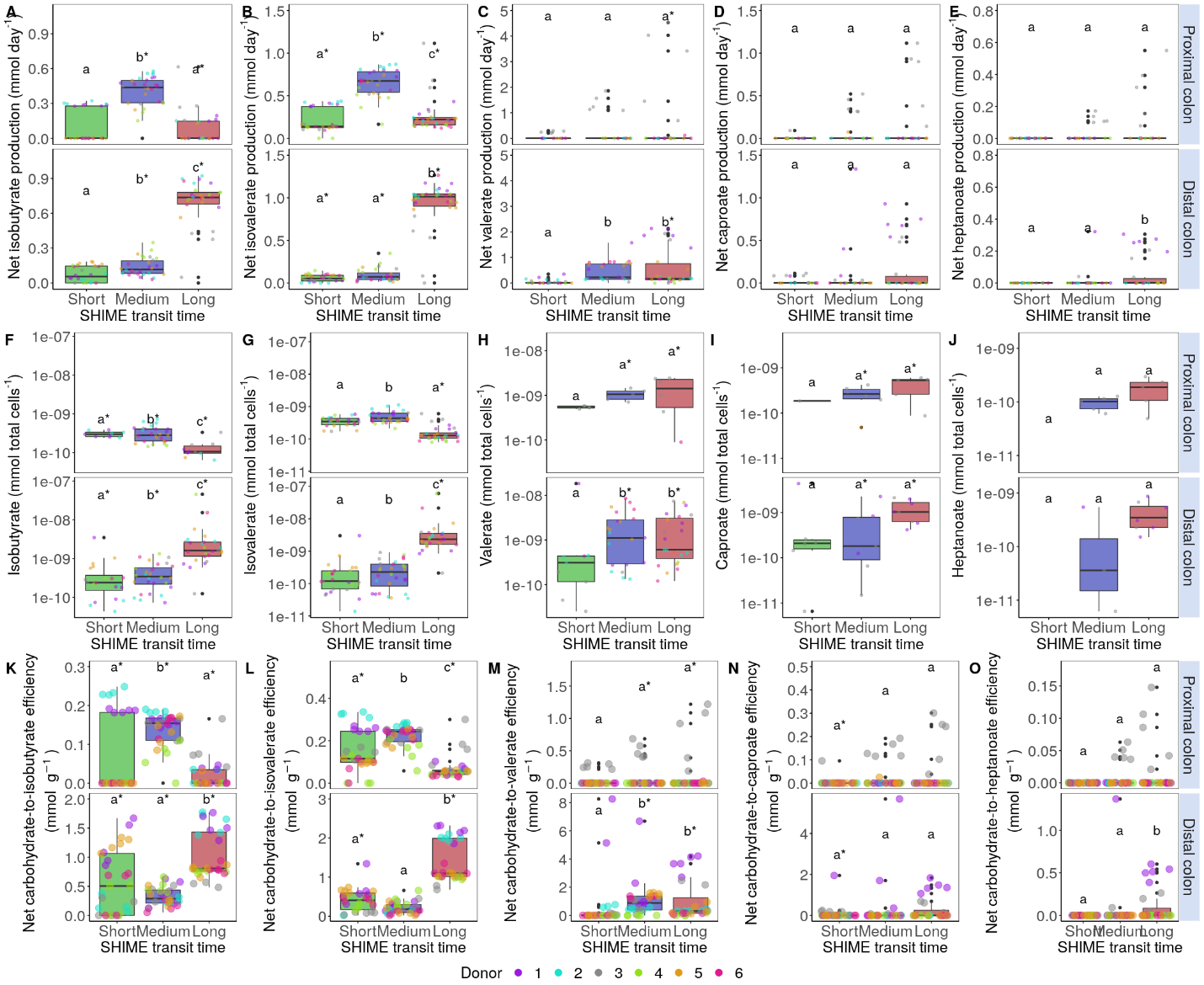


Figure S13: Net metabolite production (mmol day–1) of isobutyrate (A), isovalerate (B), valerate (C), caproate (D) and heptanoate (E) was affected by SHIME transit time (n=30). The normalised isobutyrate (F), isovalerate (G), valerate (H), caproate (I) and heptanoate (J) production relative to the total cells (mmol total cells–1) was affected by SHIME transit time (n=30). The net isobutyrate (K), isovalerate (L), valerate (M), caproate (N) and heptanoate (O) production relative to the daily carbohydrate utilisation (mmol g-1) was affected by SHIME transit time (n=30). Short, medium and long SHIME transit times were 8, 16 and 24h in the proximal colon and 13, 26 and 39h in the distal colon. Statistically significant differences between transit times are depicted by the letters a, b and c in panels A-O (unpaired two-sided Wilcoxon signed rank tests with Holm correction). Identical letters indicate no significant differences (P>0.05). Significant differences between colon regions of the same transit time are marked with asterisks (∗) (P<0.05, paired two-sided Wilcoxon signed rank tests with Holm correction). Box plots display individual data points, as well as the interquartile range, median and outliers beyond the 1.5 times interquartile range (whiskers).


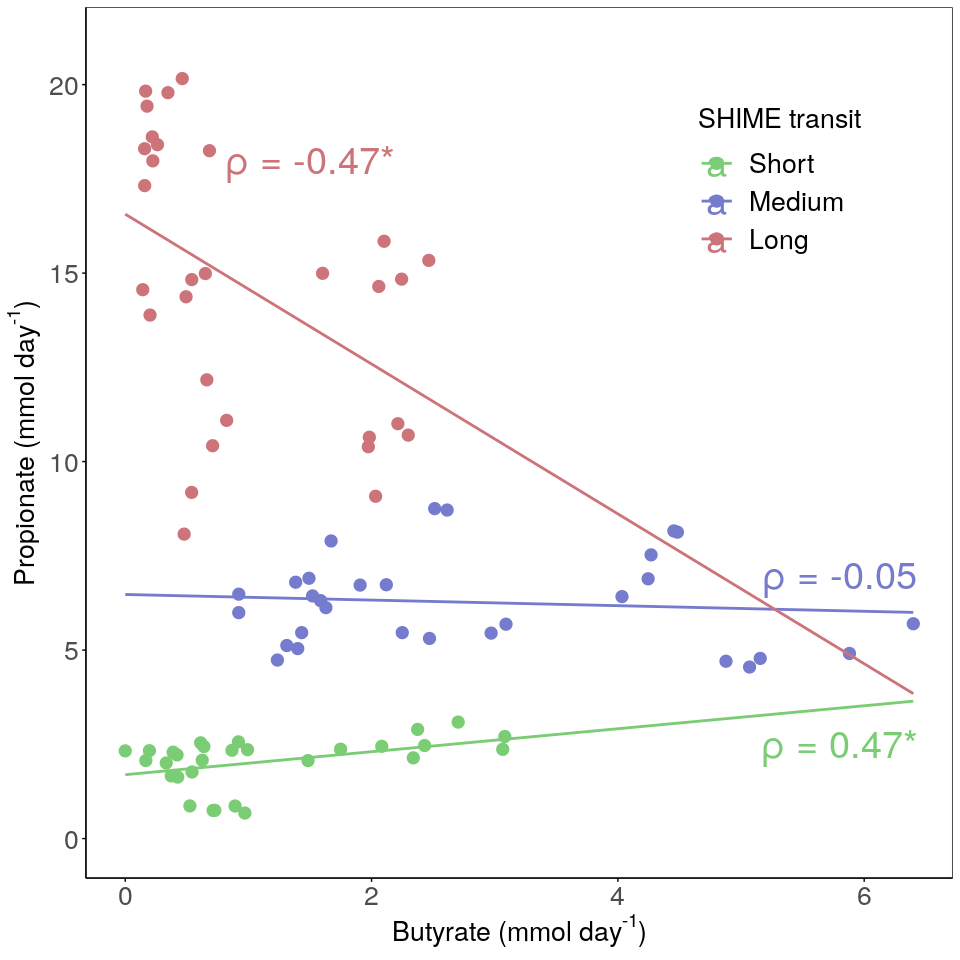


Figure S14: The net daily propionate and butyrate production correlation coefficients decreased with SHIME transit time in the proximal colon (Spearman’s rank correlation, n=30). Significant differences are marked with asterisks (∗) (P<0.05).


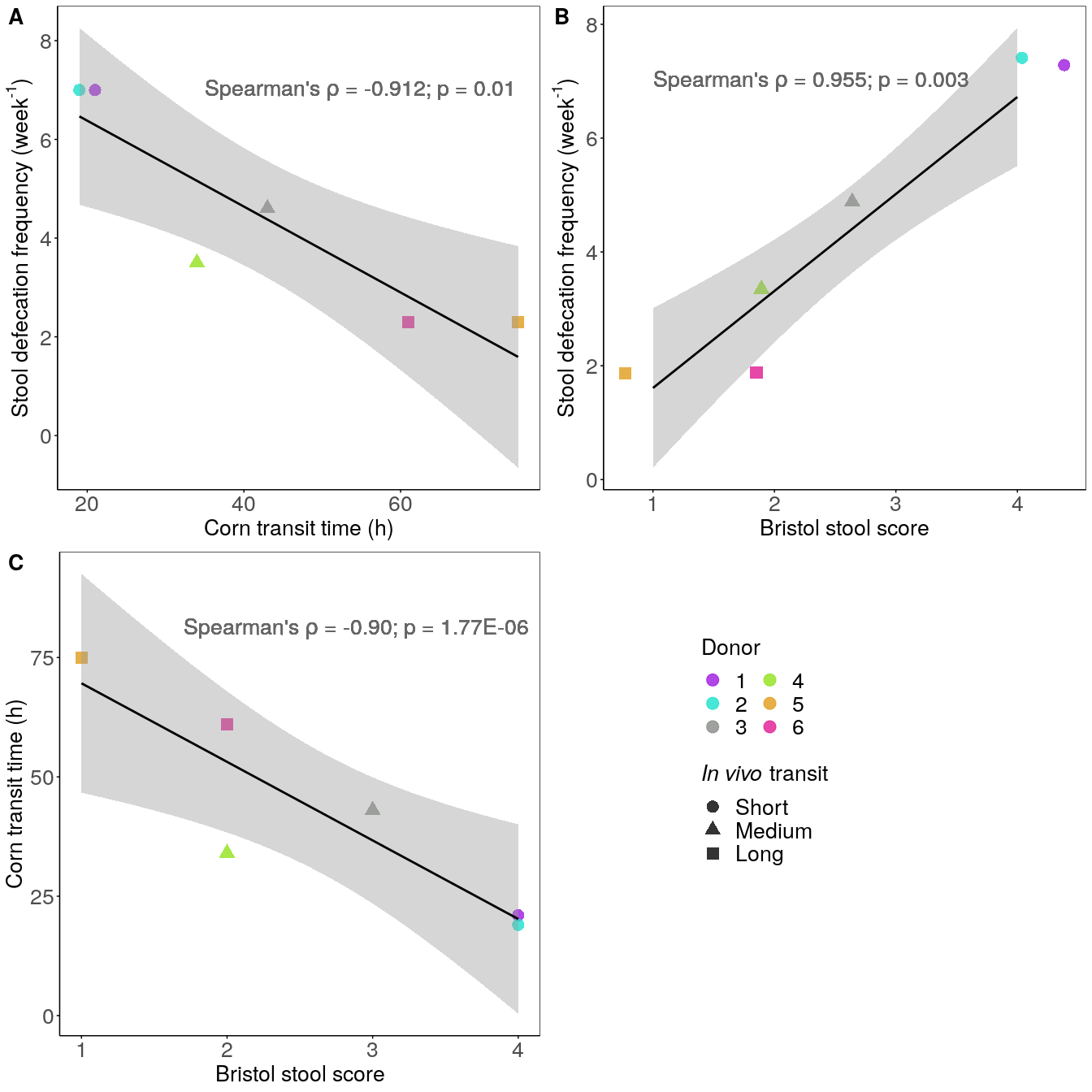


Figure S15: (**A**) The weekly stool defecation frequency (week^-1^) correlates with the corn transit time (h) (Spearman's rank correlation test). (**B**) The weekly stool defecation frequency (week^-1^) correlates with the Bristol Stool Scale (Spearman's rank correlation test). (**C**) The corn transit time (h) correlates with the Bristol Stool Scale (Spearman's rank correlation test). The *in vivo* transit time was inferred from the self-assessed corn transit time*,* stool frequency and Bristol Stool Scale (Table S1).

# Supplementary Tables

Table S1: Characteristics of the six faecal microbiome donors. *In vivo* transit times were estimated by recording the time between corn consumption and corn defecation and classified as follows: short transit ≤42h, 42h<medium transit ≤60h and long transit >60h. Sex: Male (M) or female (F). Stool frequency and corn transit time were self-assessed whereas the Bristol Stool Scale was determined by the research team.

| Donor | Sex (M/F) | Stool frequency | Estimated corn transit time (h) | Bristol Stool Scale | Transit time classification |
| --- | --- | --- | --- | --- | --- |
| 1 | M | Daily | 21 | 4 | Short |
| 2 | M | Daily | 19 | 4 | Short |
| 3 | M | Every 1.5 days | 43 | 3 | Medium |
| 4 | F | Every 2 days | 34 | 2 | Medium |
| 5 | F | Every 3 days | 75 | 1 | Long |
| 6 | F | Every 3 days | 61 | 2 | Long |

Table S2: The composition of the pancreatic juice remained constant throughout the experiment.

| Component | Composition (g L^-1^) | Manufacturer |
| --- | --- | --- |
| Oxgall dehydrated fresh bile | 6 | BD, Franklin Lakes, NJ, USA |
| Pancreatin from porcine pancreas | 0.9 | Sigma Aldrich, St. Louis, MO, USA |
| NaHCO_3_ | 12 | Carl Roth, Karlsruhe, Germany |

Table S3: Volumes of 1.5x concentrated standardised nutritional SHIME medium (17.4g L^-1^ adult L-SHIME growth medium and 6g L^-1^ corn starch, ProDigest, Zwijnaarde, Belgium), pancreatic juice (Table S2) and distilled water, added to the proximal colon each feeding cycle were adjusted to provide an equal nutrient concentration for every transit time configuration (short, medium, long). The 1.5x concentrated nutritional SHIME medium was acidified and mixed with pancreatic juice and distilled water in the stomach/small intestine compartment prior to its transfer to the proximal colon.

| Component (mL) | Short transit | Medium transit | Long transit |
| --- | --- | --- | --- |
| Nutritional SHIME feed (1.5x concentrated) | 46⅔ | 93⅓ | 140 |
| Distilled water | 133⅓ | 66⅔ | 0 |
| Pancreatic juice | 20 | 40 | 60 |
| Total volume | 200 | 200 | 200 |
|  |  |  |  |
| Influent dosage rate (L_influent_ day^-1^) | 0.60 | 0.60 | 0.60 |
| SHIME proximal colon volume (L_proximal colon_) | 0.20 | 0.40 | 0.60 |
| Influent nutrient concentration (g L_influent_^-1^) | 5.46 | 10.92 | 16.38 |
| SHIME nutrient concentration  (g L_proximal colon_^-1^) | 5.46 | 5.46 | 5.46 |
| Volumetric loading rate (g L_proximal colon_^-1^.day^-1^) | 16.38 | 16.38 | 16.38 |
| Nutrient load (g day^-1^) | 3.28 | 6.55 | 9.83 |

Table S4: Reducing anaerobic phosphate buffer (pH 6.8) was sparged and flushed with N_2_-gas prior to autoclaving.

| Component | Composition (g L^-1^) | Molar conc. (mM) | Manufacturer |
| --- | --- | --- | --- |
| L-Cystein- HCl | 1.00 | 6.34e-3 | J&K Scientific bvba, Lommel, Belgium |
| C_2_H_3_O_2_SNa | 1.00 | 8.75e-3 | Sigma Aldrich, St. Louis, MO, USA |
| NaHCO_3_ | 1.40 | 2.40e-2 | Chem-lab, Zedelgem, Belgium |
| NaCl | 0.90 | 1.07e-2 | Carl Roth, Karlsruhe, Germany |
| KH_2_PO_4_ | 6.80 | 5.00e-2 | Carl Roth, Karlsruhe, Germany |
| K_2_HPO_4_ | 8.72 | 5.00e-2 | Carl Roth, Karlsruhe, Germany |

Table S5: P values of significant differences in genus level quantitative and proportional abundances between transit times, separated by the colon regions (Kruskal-Wallis). Genera without a single significant difference in quantitative and proportional abundances between transit times were excluded from the table (P>0.05). Higher level taxa are to be interpreted as the unclassified genus belonging to the respective taxon.

|  | **Quantitative microbial community** | | **Proportional microbial community** | |
| --- | --- | --- | --- | --- |
| **Genus** | **Proximal colon** | **Distal colon** | **Proximal colon** | **Distal colon** |
| *Achromobacter* | 1.37E-07 | 1.78E-06 | 1.79E-06 | 7.84E-08 |
| *Acidaminococcus* | 1.64E-03 | 7.94E-02 | 1.32E-02 | 2.66E-01 |
| *Alistipes* | 4.63E-02 | 9.95E-07 | 4.69E-01 | 4.75E-04 |
| *Alphaproteobacteria* | 1.00E+00 | 1.23E-02 | 1.00E+00 | 1.01E-02 |
| *Anaeroglobus* | 3.15E-07 | 7.00E-05 | 2.27E-06 | 7.94E-05 |
| *Anaerostipes* | 1.42E-02 | 6.51E-04 | 4.99E-02 | 1.89E-03 |
| *Anaerotruncus* | 1.00E+00 | 4.40E-03 | 1.00E+00 | 2.04E-03 |
| *Bacillus* | 2.55E-02 | 5.81E-03 | 3.02E-04 | 2.28E-02 |
| *Bacteroides* | 7.84E-11 | 6.87E-11 | 4.63E-10 | 9.65E-05 |
| *Bifidobacterium* | 1.11E-06 | 1.56E-09 | 4.07E-09 | 3.93E-11 |
| *Bilophila* | 1.76E-05 | 9.29E-05 | 2.80E-03 | 3.06E-01 |
| *Blautia* | 1.95E-04 | 2.97E-07 | 1.26E-03 | 2.87E-01 |
| *Butyricicoccus* | 3.35E-02 | 4.07E-02 | 4.05E-01 | 6.09E-01 |
| *Butyricimonas* | 3.17E-01 | 1.43E-03 | 3.17E-01 | 1.49E-02 |
| *Cloacibacillus* | 2.49E-01 | 1.64E-03 | 2.01E-01 | 7.32E-03 |
| *Clostridiales* | 6.91E-02 | 2.41E-04 | 1.20E-01 | 1.26E-05 |
| *Clostridium_sensu_stricto* | 5.46E-03 | 6.49E-02 | 2.31E-05 | 2.74E-02 |
| *Clostridium_XlVa* | 3.61E-12 | 3.79E-05 | 1.64E-08 | 7.29E-02 |
| *Clostridium_XlVb* | 1.00E+00 | 1.26E-02 | 1.00E+00 | 1.26E-02 |
| *Clostridium_XVIII* | 1.00E+00 | 3.12E-02 | 1.00E+00 | 2.12E-02 |
| *Collinsella* | 1.71E-01 | 2.47E-10 | 2.43E-04 | 1.51E-12 |
| *Coprococcus* | 7.94E-01 | 2.93E-01 | 8.96E-01 | 3.83E-04 |
| *Desulfovibrio* | 2.85E-01 | 7.39E-05 | 1.48E-01 | 3.24E-01 |
| *Dialister* | 9.38E-11 | 3.07E-07 | 5.65E-10 | 2.29E-06 |
| *Dorea* | 2.48E-01 | 1.11E-06 | 5.43E-02 | 2.85E-09 |
| *Eisenbergiella* | 1.43E-01 | 1.67E-02 | 1.43E-01 | 3.11E-01 |
| *Enterobacteriaceae* | 3.36E-01 | 3.56E-02 | 4.00E-02 | 1.66E-03 |
| *Enterococcus* | 2.08E-02 | 1.34E-02 | 2.32E-02 | 2.52E-02 |
| *Erysipelotrichaceae* | 7.34E-01 | 2.00E-01 | 4.97E-01 | 1.05E-02 |
| *Escherichia/Shigella* | 7.84E-04 | 8.22E-01 | 9.31E-03 | 4.72E-01 |
| *Faecalibacterium* | 5.58E-01 | 1.37E-03 | 4.04E-01 | 1.46E-04 |
| *Faecalicoccus* | 7.89E-01 | 1.06E-01 | 2.85E-01 | 6.28E-03 |
| *Firmicutes* | 7.96E-01 | 3.67E-03 | 6.06E-01 | 3.06E-02 |
| *Flavonifractor* | 8.50E-01 | 5.44E-06 | 8.50E-01 | 7.76E-04 |
| *Fusicatenibacter* | 3.41E-03 | 2.08E-02 | 3.41E-03 | 1.20E-02 |
| *Intestinimonas* | 4.07E-01 | 1.50E-02 | 4.07E-01 | 1.61E-02 |

Table S5 – Continued: P values of significant differences in genus level quantitative and proportional abundances between transit times, separated by the colon regions (Kruskal-Wallis). Genera without a single significant difference in quantitative and proportional abundances between transit times were excluded from the table (P>0.05). Higher level taxa are to be interpreted as the unclassified genus belonging to the respective taxon.

|  | **Quantitative microbial community** | | **Proportional microbial community** | |
| --- | --- | --- | --- | --- |
| **Genus** | **Proximal colon** | **Distal colon** | **Proximal colon** | **Distal colon** |
| *Lachnospiraceae* | 3.09E-02 | 2.26E-05 | 2.60E-01 | 3.50E-02 |
| *Lactobacillus* | 5.32E-03 | 3.30E-01 | 3.93E-02 | 8.49E-01 |
| *Lysinibacillus* | 3.36E-02 | 3.39E-02 | 2.23E-02 | 3.39E-02 |
| *Megamonas* | 6.71E-03 | 3.38E-02 | 7.15E-01 | 4.70E-03 |
| *Mitsuokella* | 1.19E-02 | 7.15E-04 | 5.36E-05 | 2.55E-06 |
| *Murimonas* | 1.00E+00 | 1.30E-03 | 1.00E+00 | 9.19E-05 |
| *Odoribacter* | 1.00E+00 | 7.79E-03 | 1.00E+00 | 9.86E-02 |
| *Oscillibacter* | 3.17E-01 | 1.57E-04 | 3.17E-01 | 5.37E-03 |
| *Parabacteroides* | 1.56E-05 | 4.32E-12 | 1.45E-04 | 6.79E-07 |
| *Parasutterella* | 2.11E-01 | 2.38E-02 | 5.50E-01 | 6.01E-02 |
| *Phascolarctobacterium* | 3.63E-03 | 3.22E-01 | 3.77E-03 | 6.75E-01 |
| *Prevotella* | 1.85E-03 | 1.78E-02 | 6.44E-03 | 2.02E-02 |
| *Pseudoflavonifractor* | 3.17E-01 | 3.99E-02 | 3.17E-01 | 6.32E-02 |
| *Pseudomonadaceae* | 5.48E-03 | 2.58E-05 | 7.02E-01 | 1.22E-01 |
| *Pseudomonas* | 2.25E-05 | 6.49E-09 | 3.24E-01 | 1.61E-03 |
| *Roseburia* | 5.91E-03 | 4.39E-06 | 2.15E-02 | 2.22E-08 |
| *Ruminococcaceae* | 3.24E-01 | 9.06E-04 | 8.00E-02 | 8.40E-03 |
| *Ruminococcus* | 2.41E-01 | 9.70E-03 | 1.17E-01 | 3.80E-01 |
| *Stenotrophomonas* | 5.14E-07 | 8.97E-05 | 1.67E-08 | 1.85E-05 |
| *Succiniclasticum* | 1.00E+00 | 2.08E-03 | 1.00E+00 | 2.60E-03 |
| *Sutterella* | 3.00E-01 | 1.52E-06 | 1.11E-01 | 2.95E-05 |
| *Veillonella* | 2.30E-05 | 3.37E-07 | 6.07E-07 | 5.54E-08 |
| *Veillonellaceae* | 4.80E-03 | 2.65E-01 | 3.81E-03 | 2.44E-01 |
| *Victivallis* | 1.00E+00 | 1.68E-03 | 1.00E+00 | 3.88E-02 |

Table S6: RDP SeqMatch and NCBI BLAST results of the top 25 most abundant OTUs and OTUs with a proportional abundance higher than 5% within genera that responded significantly to transit time classified to the species level, as determined with 16S rRNA gene amplicon sequencing. The RDP Seqmatch similarity score (S_ab) and NCBI BLAST output of the best and next best hits are shown.

|  |  | **RDP** | **NCBI BLAST** | | |
| --- | --- | --- | --- | --- | --- |
| **OTU** | **Species** | **S_ab score** | **Query cover (%)** | **E-value** | **Identity (%)** |
| 1 | *Klebsiella pneumoniae* | #N/A | 100 | 5.00E-127 | 100 |
|  | *Enterobacter asburiae* | #N/A | 100 | 5.00E-127 | 100 |
| 2 | *Phocaeicola vulgatus* | 1.000 | 100 | 5.00E-127 | 100 |
|  | *Phocaeicola dorei* | 0.971 | 100 | 5.00E-131 | 100 |
| 3 | *Bifidobacterium adolescentis* | 1.000 | 100 | 5.00E-127 | 100 |
|  | *Bifidobacterium faecale* | 1.000 | 100 | 5.00E-127 | 100 |
|  | *Bifidobacterium longum* | 0.971 |  |  |  |
| 4 | *Megamonas funiformis* | 1.000 | 100 | 2.00E-126 | 100 |
|  | *Pectinatus frisingensis* | 0.871 | 100 | 8.00E-115 | 97 |
|  | *Megamonas rupellensis* | 0.963 |  |  |  |
| 5 | *Bifidobacterium pseudocatenulatum* | 1.000 | 100 | 5.00E-127 | 100 |
|  | *Bifidobacterium longum* | 0.946 | 100 | 5.00E-127 | 100 |
|  | *Bifidobacterium gallicum* | 1.000 |  |  |  |
| 6 | *Enterocloster bolteae* | #N/A | 100 | 5.00E-127 | 100 |
|  | *Enterocloster clostridioformis* | 0.963 | 100 | 5.00E-127 | 100 |
|  | *Enterocloster citroniae* | 0.971 |  |  |  |
| 7 | *Bacteroides kribbi* | #N/A | 100 | 5.00E-127 | 100 |
|  | *Bacteroides ovatus* | 0.972 | 100 | 5.00E-127 | 100 |
|  | *Bacteroides xylanisolvens* | 0.967 |  |  |  |
| 8 | *Bacteroides faecis* | 1.000 | 100 | 5.00E-127 | 100 |
|  | *Bacteroides thetaiotaomicron* | 1.000 | 100 | 5.00E-127 | 100 |
| 9 | *Phocaeicola massiliensis* | 1.000 | 100 | 5.00E-127 | 100 |
|  | *Phocaeicola sartorii* | 0.927 | 100 | 5.00E-122 | 99 |
| 10 | *Mitsuokella jalaludinii* | 1.000 | 100 | 2.00E-126 | 100 |
|  | *Selenomonas bovis* | 0.925 | 98 | 4.00E-118 | 98 |
| 11 | *Bacteroides fragilis* | 1.000 | 100 | 5.00E-127 | 100 |
|  | *Bacteroides finegoldii* | 0.935 | 100 | 1.00E-129 | 99 |
| 12 | *Bacteroides caccae* | 1.000 | 100 | 5.00E-127 | 100 |
|  | *Bacteroides finegoldii* | 0.874 | 100 | 1.00E-118 | 98 |

Table S6 – Continued: RDP SeqMatch and NCBI BLAST results of the top 25 most abundant OTUs and OTUs with a proportional abundance higher than 5% within genera that responded significantly to transit time classified to the species level, as determined with 16S rRNA gene amplicon sequencing. The RDP Seqmatch similarity score (S_ab) and NCBI BLAST output of the best and next best hits are shown.

|  |  | **RDP** | **NCBI BLAST** | | |
| --- | --- | --- | --- | --- | --- |
| **OTU** | **Species** | **S_ab score** | **Query cover (%)** | **E-value** | **Identity (%)** |
| 13 | *Bacteroides uniformis* | 1.000 | 100 | 5.00E-127 | 100 |
| 14 | *Pseudomonas aeruginosa* | 1.000 | 100 | 5.00E-127 | 100 |
| 15 | *Bilophila wadsworthia* | 1.000 | 100 | 5.00E-127 | 100 |
|  | *Desulfovibrio alaskensis* | 0.729 | 100 | 1.00E-98 | 93 |
| 16 | *Serratia marcescens* | 1.000 | 100 | 5.00E-127 | 100 |
|  | *Serratia nematodiphila* | 1.000 | 100 | 5.00E-127 | 100 |
| 17 | *Veillonella dispar* | 0.976 | 100 | 2.00E-126 | 100 |
|  | *Veillonella parvula* | 0.927 | 100 | 2.00E-126 | 100 |
| 18 | *Akkermansia muciniphila* | 1.000 | 100 | 5.00E-127 | 100 |
|  | *Akkermansia glycaniphila* | #N/A | 100 | 1.00E-90 | 90 |
| 19 | *Shigella flexneri* | 1.000 | 100 | 5.00E-127 | 100 |
|  | *Escherichia coli* | 1.000 | 100 | 5.00E-127 | 100 |
| 21 | *Bacteroides stercoris* | 1.000 | 100 | 5.00E-127 | 100 |
|  | *Bacteroides eggerthii* | 0.881 | 100 | 2.00E-115 | 97 |
| 22 | *Dialister hominis* | #N/A | 100 | 2.00E-126 | 100 |
|  | *Dialister propionicifaciens* | 0.922 | 100 | 2.00E-121 | 99 |
| 23 | *Parabacteroides distasonis* | 1.000 | 100 | 5.00E-127 | 100 |
| 24 | *Faecalibacterium prausnitzii* | 1.000 | 100 | 6.00E-127 | 100 |
| 25 | *Acidaminococcus intestini* | 1.000 | 100 | 2.00E-126 | 100 |
|  | *Acidaminococcus fermentans* | 0.988 | 100 | 8.00E-125 | 100 |
| 26 | *Bacteroides intestinalis* | 1.000 | 100 | 5.00E-127 | 100 |
|  | *Bacteroides cellulosilyticus* | 0.901 | 100 | 2.00E-120 | 98 |
| 29 | *Ruminococcus torques* | 1.000 | 100 | 5.00E-127 | 100 |
|  | *Mediterraneibacter massiliensis* | 0.893 | 100 | 5.00E-117 | 98 |
| 30 | *Eubacterium rectale* | 1.000 | 100 | 6.00E-127 | 100 |
| 31 | *Veillonella rogosae* | 1.000 | 100 | 2.00E-126 | 100 |
|  | *Veillonella parvula* | 0.971 | 100 | 2.00E-126 | 100 |
| 33 | *Phascolarctobacterium faecium* | 1.000 | 100 | 2.00E-126 | 100 |
|  | *Phascolarctobacterium succinatuten* | 0.842 | 100 | 5.00E-108 | 95.62 |
| 34 | *Prevotella copri* | 1.000 | 100 | 5.00E-127 | 100 |
|  | *Prevotella melaninogenica* | 0.730 | 100 | 3.00E-105 | 94.86 |
| 35 | No classification with sufficiently high enough scores (s_AB<0.9) | | | | |
| 36 | *Parabacteroides merdae* | 1.000 | 100 | 5.00E-127 | 100 |
|  | *Parabacteroides johnsonii* | 0.925 | 100 | 5.00E-122 | 98.81 |

Table S6 – Continued: RDP SeqMatch and NCBI BLAST results of the top 25 most abundant OTUs and OTUs with a proportional abundance higher than 5% within genera that responded significantly to transit time classified to the species level, as determined with 16S rRNA gene amplicon sequencing. The RDP Seqmatch similarity score (S_ab) and NCBI BLAST output of the best and next best hits are shown.

|  |  | **RDP** | **NCBI BLAST** | | | | |
| --- | --- | --- | --- | --- | --- | --- | --- |
| **OTU** | **Species** | **S_ab score** | **Query cover (%)** | | **E-value** | | **Identity (%)** |
| 38 | *Cloacibacillus evryensis* | 1.000 | 100 | | 5.00E-127 | | 100 |
|  | *Cloacibacillus porcorum* | 0.840 | 100 | | 5.00E-117 | | 98 |
| 39 | *Dialister invisus* | 1.000 | 100 | | 2.00E-126 | | 100 |
|  | *Dialister propionicifaciens* | 0.922 | 100 | | 2.00E-121 | | 99 |
| 40 | *Blautia wexlerae* | 1.000 | 100 | | 6.00E-127 | | 100 |
|  | *Blautia luti* | 0.971 | 100 | | 3.00E-125 | | 99.6 |
| 42 | *Prevotella hominis* | #N/A | 100 | | 1.00E-118 | | 98 |
|  | *Prevotella copri* | 0.819 | 100 | | 5.00E-112 | | 96 |
| 47 | *Faecalibacterium prausnitzii* | 0.980 | 100 | | 6.00E-127 | | 100 |
| 48 | *Anaeroglobus geminatus* | 1.000 | 100 | | 6.00E-127 | | 100 |
| 58 | *Clostridium symbiosum* | 1.000 | 100 | | 5.00E-127 | | 100 |
|  | *Clostridium transplantifaecale* | #N/A | 100 | | 2.00E-115 | | 97 |
| 60 | *Mediterraneibacter faecis* | 1.000 | 100 | | 5.00E-127 | | 100 |
|  | *Blautia brookingsii* | #N/A | 100 | | 2.00E-115 | | 97 |
| 61 | *Anaerobutyricum hallii* | 0.971 | 100 | | 6.00E-127 | | 100 |
| 67 | *Mitsuokella multacida* | 0.979 | 100 | | 2.00E-125 | | 100 |
|  | *Mitsuokella jalaludinii* | 0.929 | 100 | | 4.00E-118 | | 98 |
| 68 | *Clostridium scindens* | 1.000 | 100 | | 5.00E-127 | | 100 |
|  | *Dorea longicatena* | 0.837 | 100 | | 5.00E-112 | | 96 |
| 70 | No taxonomic consensus |  |  | |  | |  |
| 72 | *Ruminococcus lactaris* | 1.000 | 100 | | 5.00E-127 | | 100 |
|  | *Mediterraneibacter butyricigenes* | #N/A | 100 | | 5.00E-117 | | 98 |
| 73 | No taxonomic consensus | | | | | | |
| 74 | *Blautia luti* | 0.980 | 100 | 3.00E-123 | | 100 | |
| 76 | No taxonomic consensus | | | | | | |
| 85 | No taxonomic consensus | | | | | | |
| 103 | No taxonomic consensus | | | | | | |
| 105 | *Dialister hominis* | #N/A | 100 | | 8.00E-115 | | 97 |
|  | *Dialister propionicifaciens* | 0.829 | 100 | | 4.00E-113 | | 97 |
| 112 | *Ruminococcus torques* | 0.926 | 100 | | 1.00E-123 | | 99 |
|  | *Mediterraneibacter massiliensis* | 0.860 | 100 | | 1.00E-113 | | 97 |
| 116 | No taxonomic consensus | | | | | | |
| 129 | *Merdimonas faecis* | 0.821 | 100 | | 2.00E-110 | | 96 |
|  | *Lachnoclostridium edouardi* | #N/A | 100 | | 5.00E-107 | | 95 |

Table S6 – Continued: RDP SeqMatch and NCBI BLAST results of the top 25 most abundant OTUs and OTUs with a proportional abundance higher than 5% within genera that responded significantly to transit time classified to the species level, as determined with 16S rRNA gene amplicon sequencing. The RDP Seqmatch similarity score (S_ab) and NCBI BLAST output of the best and next best hits are shown.

|  |  | **RDP** | **NCBI BLAST** | | |
| --- | --- | --- | --- | --- | --- |
| **OTU** | **Species** | **S_ab score** | **Query cover (%)** | **E-value** | **Identity (%)** |
| 130 | *Faecalicatena orotica* | 1.000 | 100 | 5.00E-127 | 100 |
|  | *Faecalicatena contorta* | 0.930 | 100 | 2.00E-120 | 98 |
| 131 | *Cloacibacillus porcorum* | 0.947 | 100 | 5.00E-127 | 100 |
|  | *Cloacibacillus evryensis* | 0.824 | 100 | 5.00E-112 | 96 |
| 135 | No taxonomic consensus |  |  |  |  |
| 144 | *Dialister pneumosintes* | 1.000 | 100 | 2.00E-126 | 100 |
|  | *Dialister propionicifaciens* | 0.846 | 100 | 4.00E-113 | 97 |
| 147 | *Faecalicatena contorta* | 0.754 | 100 | 1.00E-108 | 96 |
|  | *Faecalicatena fissicatena* | 0.754 | 100 | 1.00E-108 | 96 |
| 149 | *Faecalibacterium prausnitzii* | 0.930 | 100 | 1.00E-126 | 100 |
| 154 | *Sellimonas intestinalis* | 1.000 | 100 | 5.00E-127 | 100 |
|  | *Ruminococcus lactaris* | 0.793 | 100 | 2.00E-105 | 95 |
| 188 | *Mediterraneibacter faecis* | 0.963 | 100 | 1.00E-123 | 99 |
|  | *Ruminococcus lactaris* | 0.898 | 100 | 5.00E-117 | 98 |
| 189 | *Megamonas funiformis* | 0.942 | 93 | 5.00E-117 | 100 |
|  | *Megamonas rupellensis* | 0.900 | 93 | 1.00E-113 | 99 |
| 191 | *Ruminococcus lactaris* | 0.872 | 100 | 5.00E-112 | 96 |
| 198 | *Veillonella dispar* | 0.910 | 98 | 4.00E-118 | 98 |
|  | *Veillonella parvula* | 0.861 | 100 | 4.00E-118 | 98 |
| 214 | *Prevotella marseillensis* | #N/A | 100 | 5.00E-127 | 100 |
|  | *Prevotella hominis* | #N/A | 100 | 1.00E-88 | 91 |
|  | *Prevotella copri* | 0.660 | 100 | 5.00E-87 | 91 |
| 230 | *Roseburia faecis* | 0.935 | 100 | 6.00E-127 | 100 |
| 236 | *Mitsuokella jalaludinii* | 0.917 | 97 | 8.00E-115 | 98 |
|  | *Selenomonas bovis* | 0.842 | 96 | 2.00E-106 | 96 |
| 253 | *Lacrimispora indolis* | 0.832 | 100 | 2.00E-115 | 97 |
|  | *Lacrimispora amygdalina* | 0.852 | 100 | 2.00E-115 | 97 |
| 254 | *Bilophila wadsworthia* | 0.933 | 100 | 1.00E-118 | 98 |
|  | *Desulfovibrio alaskensis* | 0.663 | 100 | 2.00E-90 | 91 |
| 274 | *Tyzzerella nexilis* | 0.963 | 100 | 5.00E-127 | 100 |
|  | *Faecalicatena fissicatena* | 0.881 | 100 | 1.00E-118 | 98 |
| 440 | *Enterocloster clostridioformis* | 0.815 | 100 | 1.00E-113 | 97 |
|  | *Enterocloster citroniae* | 0.823 | 100 | 5.00E-112 | 96 |
